# Application of a Bayesian Network Learning Model to Predict Longitudinal Trajectories of Executive Function Difficulties in Elementary School Students

**DOI:** 10.3390/jintelligence10040074

**Published:** 2022-09-23

**Authors:** Eun-Kyoung Goh, Hyo-Jeong Jeon

**Affiliations:** 1 Human Life Research Center, Dong-A University, Saha-gu, Busan 49315, Korea; 2 Department of Child Studies, Dong-A University, Saha-gu, Busan 49315, Korea

**Keywords:** executive function difficulties, Bayesian network learning, elementary school students

## Abstract

Executive function is the mental ability to modulate behavior or thinking to accomplish a task. This is developmentally important for children’s academic achievements and ability to adjust to school. We classified executive function difficulties (EFDs) in longitudinal trajectories in Korean children from 7 to 10 years old. We found predictors of EFDs using latent class growth analysis and Bayesian network learning methods with Panel Study data. Three types of latent class models of executive function difficulties were identified: low, intermediate, and high EFDs. The modeling performance of the high EFD group was excellent (AUC = .91), and the predictors were the child’s gender, temperamental emotionality, happiness, DSM (Diagnostic and Statistical Manual of Mental Disorders) anxiety problems, and the mother’s depression as well as coparenting conflict recognized by the mother. The results show that using latent class growth analysis and Bayesian network learning are helpful in classifying the longitudinal EFD patterns in elementary school students. Furthermore, school-age EFD is affected by emotional problems in parents and children that continue from early life. These findings can support children’s development and prevent risk by preclassifying children who may experience persistent EFD and tracing causes.

## 1. Introduction

### 1.1. The Importance of Executive Function Development in School-Age Children

Executive function (EF) is defined as the mental functions of planning, organizing, exploring, and controlling impulses in order to achieve goals (Carlson et al. 2004; Welsh et al. 1991). Executive function difficulties (EFDs) occur when people have trouble controlling their thoughts and actions in the context of achieving goals or solving problems. In this study, EFDs relate to planning–organizing, behavior control, emotional control, and attention–concentration difficulties (Song 2014).

Miyake and Friedman (2012) suggested that individual differences in EF show: both unity and diversity, with inhibition being a key requirement of common EF, substantial genetic contributions, clinical and societal relevance, and developmental stability. In this study, we also assume that the core characteristic of EF is the ability to self-regulate to achieve goals. Individual differences in EF are, on the one hand, influenced by factors with a biological basis, such as temperament (Leve et al. 2013), and, on the other hand, they can be influenced by contextual factors, such as parenting (Fay-Stammbach et al. 2014). It is necessary to consider congenital and contextual factors because of the complexity of EF-related variables to understand the characteristics of EF development (Zelazo 2013). In general, EF develops rapidly in early childhood and slowly thereafter (Center on the Developing Child at Harvard University 2011). Elementary school children are expected to show a relatively gradual developmental trajectory; however, it is assumed that there are individual differences in the level of EFD. Therefore, knowing the predictors of EFD may help to select groups in need of early support.

EF is one of the most important mental functions school-age children need for a successful academic performance and school life. Although intelligence is the most powerful predictor of children’s academic achievement (Mayes et al. 2009; Roth et al. 2015), EF and intelligence positively contribute to overall academic achievement (Yeniad et al. 2013). Likewise, EFDs consistently predict elementary students’ academic performance (Goh 2020) and negatively predict overall school adaptation, which includes peer relationships and abiding by school rules (Goh and Jeon 2020). However, the EF that predicts childhood intelligence is an undifferentiated unity (Brydges et al. 2012). In this study, in the context of elementary school, EFD subfactors are not differentiated, and the overall EFD trajectory is traced.

### 1.2. Child-Related Predictors of EF Development

The identification of factors predicting developmental differences in EF or EFD can contribute to the development of interventions that consider individual differences and the classification of children in need of intervention. The main factors predicting longitudinal EF or EFD development include congenital characteristics, parental influence, and characteristics of the home environment. A child’s EF can be related to innate characteristics, such as gender differences and temperament, and psychological behavioral characteristics, such as behavioral problems, self-esteem, and feelings of well-being. In relation to gender differences before school age, girls may be better than boys in inhibition (Ardila et al. 2005; Klenberg et al. 2001), but the EF of Brazilian school-age children did not differ according to gender (Jacobsen et al. 2017). Gender is not an important predictor of EF (Grissom and Reyes 2019), but if the effects of gender difference vary by period, it is necessary to examine whether they are related to longitudinal EFD changes. Temperament is a genetic personality trait that appears early in life (Buss and Plomin 2014). Temperamental difficulties are associated with low executive cognitive function as well as aggressive and antisocial behavior (Giancola et al. 1998). Particularly, the negative emotionality of temperament has an important influence on the early development of EF (Leve et al. 2013). Therefore, it is necessary to investigate whether the influence of children’s innate gender differences and temperament predicts EFD trajectories during elementary school.

Longitudinal EF changes in children are related to behavioral and psychological difficulties, with longitudinal relationships between children’s EFD and internalizing (Wang and Zhou 2019) and externalizing (Tillman et al. 2015) behavioral problems. EFD has been shown to affect emotional symptoms, hyperactivity, conduct/peer problems, and academic competence (Hughes and Ensor 2011). EFD-driven behavioral problems can negatively affect a child’s self-esteem and well-being, affecting EFD in turn. Low self-esteem due to adaptation difficulties heightens fear of rejection and failure and complicates EF operation (He et al. 2021; Tillman et al. 2015). The child’s EFD and happiness become a reciprocal longitudinal relationship (Sung and Choi 2021). However, it is necessary to examine the relationship between EFD and various behavioral problems.

### 1.3. Parent-Related Predictors of EF Development

Parental predictors that affect early EF development include parental psychological factors, interactions, and parenting behaviors. First, depression, a psychological factor for parents, has a negative effect on EF development (Hughes et al. 2013). In particular, maternal or perinatal maternal depression negatively affects EF in children (Park et al. 2018; Power et al. 2021). Parenting stress also impedes preschool children’s EF development (De Cock et al. 2017). Exposure to marital conflict and family violence (Gustafsson et al. 2015), parent–child interactions (Rhoades et al. 2011), and levels of family cohesion and organization (Schroeder and Kelley 2009) are also related to behavioral control or metacognition in children. Finally, regarding parenting style, if the mother is insensitive and the father harsh, it will have a negative effect on the child’s EF development (Lucassen et al. 2015). Conversely, the mother’s attention-oriented parenting behavior (Conway and Stifter 2012) and positive parenting behavior (Bernier et al. 2012; Spruijt et al. 2018) positively impact children’s EF development. The father’s good parenting qualities and coparenting (Altenburger and Schoppe-Sullivan 2021) are also associated with the child’s inhibitory control. These parenting behaviors have a longitudinal effect on their children’s EF (Bernier et al. 2010; Meuwissen and Englund 2016). Among the factors involved in the various parenting contexts, family interactions affect EF more immediately (Hughes and Ensor 2009) and have a longitudinal effect (Hughes et al. 2020). In addition, since parent–child communication is also affected by parenting stress and depression (Ponnet et al. 2013), it is necessary to structurally analyze the process by which these factors affect the development of children’s EFD. However, studies examining the relationship between parental variables and children’s EF have mainly focused on preschool children. Given the importance of EF in school life, it is necessary to determine whether negative parental influences on their children’s EFD persist throughout school age.

### 1.4. Home-Related Predictors of EF Development

Various factors in the home environment can also affect a child’s EF. First, a low family income makes it difficult for caregivers to focus on parenting (Conger et al. 2002). The resulting low-quality parenting environment can negatively impact a child’s EF development. Global surveys, which include developed and developing countries, show a strong correlation between socioeconomic status (SES) and childhood EF (Fernald et al. 2011). SES affects EF not only in children but also in other age groups (Last et al. 2018). An environment rich in learning stimuli, such as books, has a positive effect on the development of children’s EF (Dilworth-Bart et al. 2007; Sarsour et al. 2011). Abundant stimuli at home provide children with diverse experiences and promote interaction between parents and children (Kim et al. 2012). As such, the home environment can include environmental stimuli and parenting behaviors (Lee and Kwak 2008). The HOME tool was developed to investigate the relationship between the quality of the home environment and child development (Bradley and Caldwell 1979). It has shown that children’s cognitive ability and behavioral development were positively affected by the structure of the home environment, cognitive stimulation, emotional support, and sensitive parenting behavior (Cole 2005; Kim and Kwak 2015). Therefore, understanding how various aspects of the home environment contribute to a child’s EF development can be helpful for providing specific support for children.

### 1.5. Developmental Changes in EF

EF development occurs throughout life; in particular, the developmental patterns of EF and self-regulation in early childhood develop more rapidly than in other periods (Center on the Developing Child at Harvard University 2011). However, the pattern of the EF’s developmental trajectory may vary according to individual differences (Goh et al. 2012). To support EF development in children, it is necessary to classify the longitudinal trajectories of children’s EFD and predict risk factors.

Although factors influencing EF vary widely, there is no study to classify the longitudinal trajectories of children’s EF and to find the risk factors considering all predictors, such as child-related, parent-related, and home-related predictors together, because of the limitation of the analysis method and the longitudinal data. Bayesian network learning (BNL) helped us to deal with all predictors and find out the risk factors of executive function difficulties using the Panel Study on Korean Children.

Bayesian networks allow us to visually represent relationships between variables without controlled experiments and determine whether there is a causal relationship from the data (Koski and Noble 2009). However, the Bayesian network approach has been used more as a meta-analysis method than for predicting child development risks or finding a predictor and using it as a basis for preventive intervention (Sitnik-Warchulska et al. 2021). It is possible to find predictors that continuously cause EFD through BNL; therefore, this study classified longitudinal EFD patterns in elementary school children by applying latent class growth analysis (LCGA) and BNL. Finally, we evaluated the performance of these classification models and identify predictors that influence EFD, such as behavioral problems and parenting behavior. These analyses will serve as a basis for diagnosing difficulties in children experiencing EFD throughout their school years and providing support for preventive interventions.

This study was activated by two research questions:

1. What are the characteristics and incidences of EFD longitudinal trajectory patterns?
2. What are the characteristics of the predictors associated with EFD trajectory patterns?

## 2. Materials and Methods

### 2.1. Procedure

**Figure 1:**
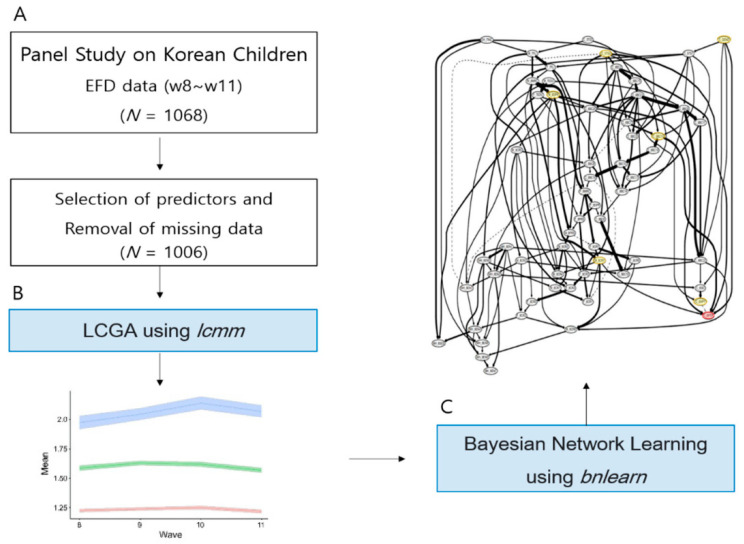
Schematic view of the methodology. (**A**) As a sample selection step, samples with no missing EF longitudinal data and predictors were selected for four years, after which samples with missing values were removed. (**B**) The longitudinal latent layer of EF was confirmed using the lcmm package. The pink line is class 1, the green line is class 2, and the blue line is class 3. (**C**) Using the BNL algorithm with the bnlearn and bootnet package, probabilistic prediction models for classifying EF latent layers were developed, and the performances of the classification models were compared and analyzed. The red circles are the Effect nodes (C_EFPT) of model 4 (threshold = .85) and the yellow circles are the parent nodes (C_GEND, C_5TS2, C_8HPY, C_8BC10, M_8DPR, M_8CR7).

The goal of this study is to utilize LCGA and BNL to compare the longitudinal patterns of EFD and to improve the classification of groups with different patterns. The main predictors contributing to this classification were identified, and the probabilistic direction between the predictors was estimated. The Panel Study on Korean Children (PSKC) is a representative panel survey conducted by the Korean government. The Korea Institute of Child Care and Education (KICCE) has been preparing this survey since 2006. Children born in Korea between April and July 2008 will be followed until they reach adulthood, and data will be collected with the consent of the respondents (KICCE 2008). The PSKC data can be freely used for academic purposes by researchers who have identified their affiliations. We acquired data by following the required procedures. Figure 1 depicts the methodology.

### 2.2. Participants

Participants were children born between April and July 2008, who entered elementary school in 2015. The analyzed data came from the 5th to 11th PSKC surveys, conducted between 2012 and 2018. The sample size was 1006, with no omissions in the variables of interest. The sample comprised 504 boys (50.1%) and 502 girls (49.9%). More than 70% of parents had a college degree or higher (73.6% of fathers and 72.5% of mothers). The average age of mothers was mid-30s (*M* = 36.7, *SD* = 3.6), and the average age of fathers was late 30s (*M* = 39.2, *SD* = 3.9).

### 2.3. Measures

#### 2.3.1. Children’s Executive Function Difficulty

To analyze the longitudinal pattern of EFD from grades 1 to 4 of elementary school, we used data collected from the 8th to 11th rounds of the PSKC. EFD was measured using Song’s (2014) Executive Function Difficulty Screening Questionnaire (EFDSQ), developed to self-assess the extent to which children and adolescents experience EFD, and measured by subjective reporting. It was validated through a significant correlation with the Stroop test result (Song 2014). The EFDSQ used in the PSKC was modified to allow the child’s mother to evaluate it, and contains 40 items: planning and organization difficulties (11 items), behavior regulation difficulties (11 items), emotion regulation difficulties (eight items), and inattention (10 items). Responses were recorded on a three-point Likert scale ranging from 1 to 3, with higher scores indicating greater difficulty in EF. The Cronbach’s alpha for the EFD was .94 between the 8th and 10th waves and .95 at the 11th wave.

#### 2.3.2. Child-Related Predictors of EFD Developmental Changes

Temperament. The data on children’s temperament were collected in the 5th survey of the PSKC using the Emotionality, Activity, Sociability (EAS) Temperament Survey for Children (Buss and Plomin 2014; Mathiesen and Tambs 1999). The EAS is a five-point Likert scale with 20 items consisting of three subdomains (emotionality, activity, and shyness/sociability). In the PSKC data, EAS was assessed by the child’s mother, and the higher the score, the stronger the subdomain characteristics of temperament. The Cronbach’s alphas for emotionality, activity, and sociality were .75, .76, and .83, respectively.

Self-Esteem. The data on children’s self-esteem were collected using PSKC’s 8th survey as a tool modified by the Millennium Cohort Study (MCS 2012) to allow children to respond to the Rosenberg Self-Esteem scale (Rosenberg 1965). It is a four-point Likert scale with five items, and the higher the score, the better the child’s self-esteem. The Cronbach’s alpha for self-esteem was .74.

Happiness. The data on children’s happiness were collected using the MCS scale (MCS 2012) in the 8th PSKC survey. Children responded according to their feelings on a four-point Likert scale on six items (schoolwork, the way they look, family, friends, school, and their life as a whole). The higher the score, the greater the child’s sense of happiness. The Cronbach’s alpha for happiness was .74.

Behavioral problems. The data on the children’s behavioral problems were collected by parents and assessed at the 8th PSKC survey using the Korean Child Behavior Checklist (K-CBCL) (Oh and Kim 2010), standardized for Korea from the Child Behavior Checklist (CBCL) (Achenbach and Rescorla 2001). The problem behavior variables of children in this study were all 18 K-CBCL subscales. In the original study, K-CBCL items were rated on a three-point Likert scale; in the current study, the T-scores were calculated and used for analysis instead of just the raw scores of each scale. The Cronbach’s alphas for total problem behavior, internal problem behavior, and external problem behavior were .92, .80, and .85, respectively.

#### 2.3.3. Parent-Related Predictors of EFD Developmental Changes

Parental Depression. The data on parental depression were collected from the 8th survey of the PSKC using the six-item Kessler scale (Kessler et al. 2002). This tool was reported by each parent on a five-point Likert scale, with a higher score indicating a higher degree of depression. The Cronbach’s alpha for the mother’s depression was .93, and for the father’s it was .94.

Parental Stress. The data on parental stress were collected from the PSKC’s 8th survey using 11 items that correspond to “pressures pertaining to the parental role and distress” (Kim and Kang 1997), adapted from the Parenting Stress Index-Short Form (Abidin 1990). Parents responded on a five-point Likert scale with higher scores indicating higher parenting-related stress. The Cronbach’s alpha for the mother’s parenting stress was .90, and for the father’s it was .88.

Marital Conflict. The data on marital conflict were collected in the 8th PSKC survey by modifying the Prevention and Relationship Enhancement Program tool (Markman et al. 2001). The revised tool was reported by each parent on a five-point Likert scale with eight items. A higher score indicates a higher level of marital conflict. The Cronbach’s alpha for the mother’s marital conflict was .92, and for the father’s it was .91.

Family Interaction. The data on family interaction were collected from the 8th PSKC survey by selecting only items from the FACES IV manual (Olson 2010) corresponding to balanced cohesion and balanced flexibility. Each parent assessed family interactions through a total of 14 items on a five-point Likert scale. A higher score indicates that the family has healthy cohesion and flexibility. The Cronbach’s alpha for the mother’s family interaction was .91, and for the father’s family interaction it was .92.

Parenting Behaviors. The data on parenting behaviors were produced by PKSC researchers with reference to the Korean Parenting Style tool (Cho et al. 1999) and collected in the 8th survey of the PSKC. The parenting behavior scale used in this study consists of six items corresponding to warm parenting behavior and six items corresponding to controlled parenting behavior. Each parent assessed the items using a five-point Likert scale, and the higher the score, the stronger the characteristics of either of the two parenting styles. The Cronbach’s alpha for the mother’s warm parenting behavior was .87, and for the father’s warm parenting behaviors it was .87. The Cronbach’s alpha for the mother’s controlled parenting behavior was .75, and for the father’s controlled parenting behavior it was .78.

Coparenting. The data on coparenting were collected from the 8th survey using McHale’s scale (McHale 1997), which PSKC researchers translated and modified. The scale consists of four subfactors: family integrity (seven items), disparagement (three items), conflict (two items), and reprimand (four items). Each parent reported on a seven-point Likert scale of 16 items, with a higher score indicating a higher level of each subfactor. The Cronbach’s alpha of coparenting evaluated by parents was low, at about .50.

Parent–Child Interaction. The data on parent–child interaction were used after translating some of the parent–child interaction items among the Home Environment, Activities, and Cognitive Stimulation Questions (HEQs) used by the Early Childhood Longitudinal Study Kindergarten Cohort (ECLS-K). The data were collected from the 8th PSKC survey, and each parent answered nine questions using a four-point Likert scale. A higher score indicates a higher level of parent–child interaction. The Cronbach’s alpha for the mother’s parent–child interaction was .84, and for the father’s parent–child interaction it was .87.

#### 2.3.4. Home-Related Predictors of EFD Developmental Changes

The factors corresponding to the quality of the home environment are the parents’ educational level, monthly household income, subjective SES, and Middle Child-HOME (MC-HOME). Parental education level was collected by each parent, responding as follows: the household’s monthly income was an open-ended question, answered in units of KRW 10,000 (Korean won currency); educational level and monthly income were collected from the PSKC’s 7th survey, which had the fewest missing values; subjective SES was measured by presenting a ladder symbolizing SES levels to the caregiver and asking them to indicate their SES on the ladder. A higher score indicates a higher subjectively perceived SES.

The MC-HOME (Caldwell and Bradley 2003) was translated and used by PSKC researchers to evaluate the quality of the home environment for raising children. MC-HOME was assessed by responses from parents raising children directly. It had a total of 59 items: Responsivity (10 items), Encouragement of maturity (7 items), Emotional climate (8 items), Learning materials and Opportunities (8 items), Enrichment (8 items), Family companionship (6 items), Family integration (4 items), and Physical environment (8 items). In this study, the results of caregivers’ responses to this tool were converted to binary data (0;1), with a higher score indicating a better home environment. The Cronbach’s alpha for MH-HOME was .86. Many variables were included in the current analysis and are presented in the Supplementary Materials as abbreviated codes measured by time period (Table 1).

### 2.4. Statistical Analyses

#### 2.4.1. Latent Class Growth Analysis

LCGA was performed to derive the latent group model of the longitudinal trajectory of EFD until the first grade of elementary school children reached the fourth grade. The goal of LCGA is to develop and analyze a model (the fixed-effect model) that assumes the homogeneity of individual data within a specific latent group in tracing longitudinal data and fixed variance and covariance (Wardenaar 2020). The following criteria were used to determine the number of suitable latent layers. First, the number of suitable latent layers was confirmed using Akaike Information Criterion (AIC), Bayesian Information Criteria (BIC), Sample-Size-Adjusted Bayesian Information Criteria (SABIC), and entropy. Second, only models in which the classified group size was greater than 1.0% of the total sample were compared, and the final model was selected. In this study, LCGA was performed using the lcmm package in R.

#### 2.4.2. One-Way ANOVA and Spearman’s Rank Correlation Analysis

A one-way analysis of variance (ANOVA) and Spearman’s rank correlation analysis were performed to understand the characteristics of the latent group of the EFD longitudinal trajectory pattern and predictive variables; 58 predictors were transformed into z-values and applied to all analyses. The z-values of the predictor variable and the correlation result are shown as a heat map. Three EFD longitudinal trajectory patterns (C_EFPT) were set as one ordinal variable in the order of high risk. The C_EFPT was put into Spearman’s rank correlation analysis along with the predictors.

#### 2.4.3. Bayesian Network and xgBoosting Learning

BNL was utilized to infer causal relationships with observed data. The reasoning process consisted of two steps: learning the causal structure from data and estimating the parameters of the variable to be predicted based on the structure. First, in structure learning, the causal relationship between variables was estimated as conditional probability using Bayes theorem and visualized as a graph called Directed Acyclic Graphs (DAGs). Next, in parameter learning, the variables constituting the nodes of the DAG were input as continuous variables in this study. Therefore, the estimation of the response variable (C_EFPT) was expressed as a linear regression of the explanatory variables. As a result, the final performance evaluation for the EFD risk in this study was also calculated by the regression equation.

The Bayesian network consists of one DAG, where nodes in the DAG represent the corresponding random variables, and the directed edges represent dependencies between variables. At this directed edge, the node from which the direction originates becomes the parent node, and the node that arrives becomes the child node (Ben-Gal 2008). For any set of random variables, the joint probability density function is formulated as a multiplication of individual density functions, given the parent variables (Russell and Norvig 2003).

$$p\left(x\right)=\underset{v\in V}{\prod }p(x_{v}|x_{\mathrm{pa}\left(v\right)})$$

where $x\left(x_{v}\right),\;v\;\in V$ is a set of random variables indexed by V, and pa(v) is the set of parent variables of V.

We designed a network structure with a binary variable effect model for the EFD longitudinal trajectory. In the EFD longitudinal trajectory patterns (C_EFPT), the relatively high-risk group was 1 (positive) and 0 (negative). Three EFD longitudinal trajectory patterns were estimated in the LCGA performed before BNL. Accordingly, the final effect is expressed as a discrete variable and modeled in five ways.

#### 2.4.4. Evaluating Learning Model

In the Bayesian network structure, the child node of the effect node is no longer created because the effect node is assumed to be the final child node. Accordingly, we blacklisted all edges originating from the effect node so that no edges in that direction were generated. In addition, we included edges with directions from the future to the past and from acquired to innate in the blacklist. In this study, we used the R-packages bnlearn and bootnet for bootstrap-based inference (Bootstrapping = 2000).

We trained the linear regression algorithm estimated by BNL on a dataset corresponding to 70% of the total data (N = 1006) and tested it on the remaining 30% of the dataset. A data split was randomly performed to regularize modeling, and ten-fold cross-validation was performed using the caret package in all processes. The area under the receiver operating characteristic curve (AUC) was calculated using the pROC package. The accuracy, sensitivity, and specificity were calculated using the following formulae:

$$\mathrm{Accuracy}=\frac{\left(\mathrm{True}\;\mathrm{Positive}+\;\mathrm{True}\;\mathrm{Negative}\right)}{\left(\mathrm{True}\;\mathrm{Positive}+\mathrm{False}\;\mathrm{Negative}+\mathrm{False}\;\mathrm{Positive}+\;\mathrm{True}\;\mathrm{Negative}\right)}\;\mathrm{Sensitivity}=\frac{\mathrm{True}\;\mathrm{Positive}}{\left(\mathrm{True}\;\mathrm{Positive}+\mathrm{False}\;\mathrm{Negative}\right)}\;\mathrm{Specificity}=\frac{\;\mathrm{True}\;\mathrm{Negative}}{\left(\mathrm{True}\;\mathrm{Negative}+\mathrm{False}\;\mathrm{Positive}\right)}$$

To compare the performance of BNL and other machine learning classifiers, the pattern of the longitudinal trajectory of children’s EF difficulties was predicted using the xgBoost algorithm (Dey 2016), which is known to have a very high predictive performance. The performance evaluation of modeling xgBoost using the xgboost package was performed in the same way as the performance evaluation process of Bayesian network modeling. A grid search strategy was used to identify the best combination of hyperparameters using the caret package for the xgBoosting model. All statistical analyses were performed using version 4.0.5 of the R software (R Core Team 2020). The statistical significance level was set at .05. Finally, while analyzing the Bayesian network structure, predictors that become the parent node of the effect node (C_EFTP) were identified and compared with the variable with a high contribution to xgBoosting. In BNL and xgBoosting modeling, all predictors were input-normalized to z-values. The DMwR package was used to ensure the sample sizes of groups were similar and placed into models for data balancing of the imbalanced models (Table 4, Models 2 to 5). The analysis codes and graphics produced are provided as supplements.

## 3. Results

### 3.1. Characteristics and Incidence of EFD Longitudinal Trajectory Pattern

**Figure 2:**
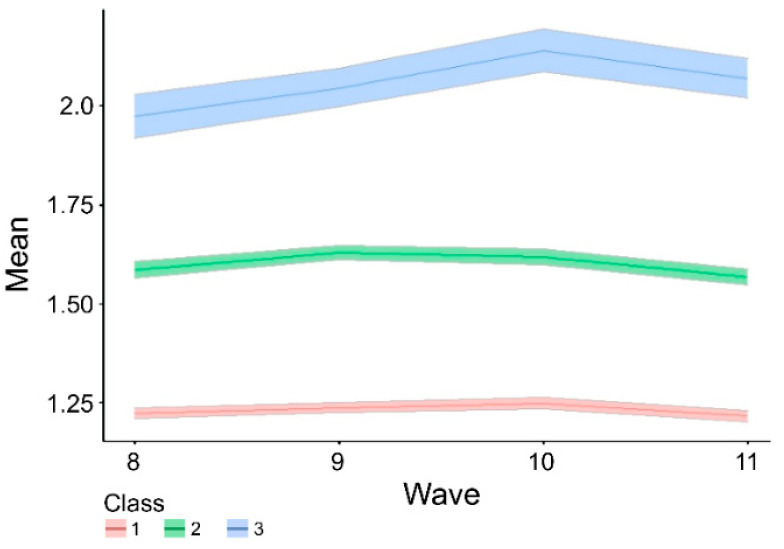
The mean and 95CI in EFD trajectory. For each wave 8–11 period, the mean of the EFD of the three latent layers and the confidence interval (Bootstrapping = 1000, 95% CI) by Bootstrapping are graphed. At all times, the mean of class 1 is the smallest, and the mean of class 3 is the largest.

Using the LCGA method, the model with three layers was found to be the most suitable. In the model, the group with the highest EF difficulty (class 3) had the lowest proportion (8.65%). Children in class 1 rarely experience EF difficulties on average, and children in class 3 occasionally experience EF difficulties on average (Table 2 and Table 3, Figure 2).

### 3.2. Characteristics of Predictors Associated with EFD Trajectory Patterns

#### 3.2.1. Differences of Predictors

**Figure 3:**
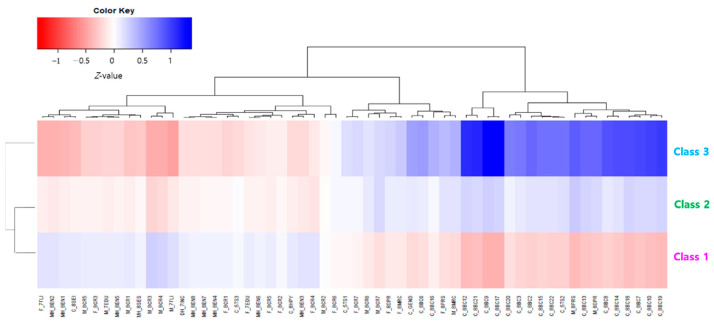
Differences of predictor’s z-values between EFD trajectory patterns.

The z-value of predictors for each latent group of the EFD longitudinal trajectory pattern was visualized and is presented in Figure 3. The z-values of most predictors were significantly different between the latent groups (*p* < .05).

Class 1, which had the lowest EFD, had a positive relationship with variables related to the home environment and family interaction but a negative relationship with variables, such as child behavioral problems, parenting stress, parental depression, and marital conflict.

#### 3.2.2. Spearman’s Rank Correlation

**Figure 4:**
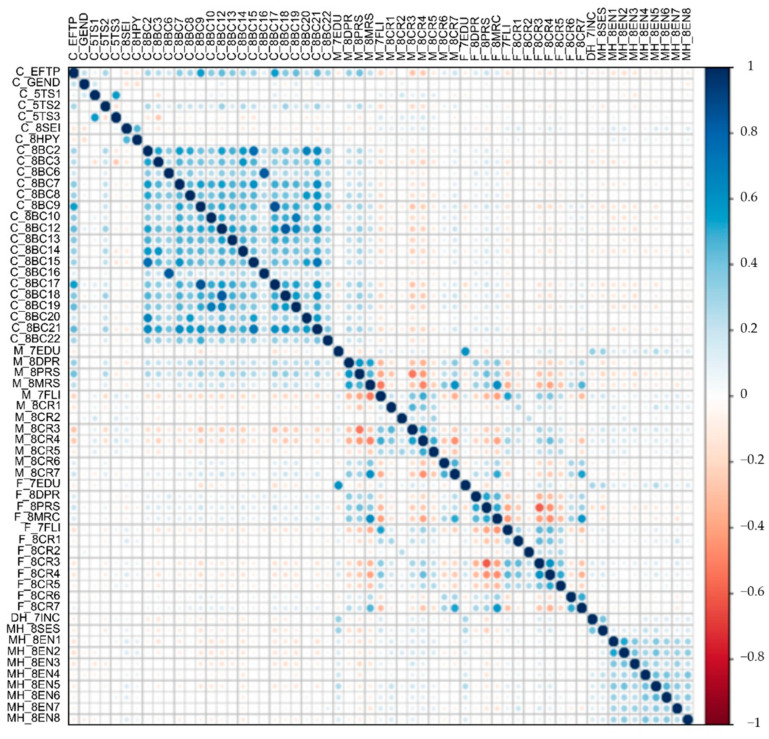
Spearman’s rank correlation analysis. When the risk of children’s EFD is taken as the ordinal variable, the larger the circle, the greater the probability of rejecting the null hypothesis of the rank correlation. The darker the blue, the greater the positive correlation, and the darker the red, the greater the negative correlation.

Three EFD longitudinal trajectory patterns were set as one ordinal variable (C_EFPT) in the order of high risk, and C_EFPT was put into Spearman’s rank correlation analysis along with the factors in Figure 3. First, in terms of child factors, gender, temperament, and behavioral problems have a rank correlation with C_EFPT. The degree of correlation with C_EFPT for gender is small if any relationship does exist in the criteria (Rovai et al. 2014). The temperament subfactors have little or no relationship with C_EFPT, but negative emotionality has a relatively high correlation with temperament factors (Figure 4).

Most of the behavioral problems, such as aggressive behaviors (*r_s_* = .35, *p* < .001), other problems (*r_s_* = .30, *p* < .001), DSM anxiety problems (*r_s_* = .35, *p* < .001), DSM attention-deficit/hyper activity problems (*r_s_* = .43, *p* < .001), DSM oppositional defiant problems (*r_s_* = .35, *p* < .001), withdrawn/depressed (*r_s_* = .32, *p* < .001), rule-breaking behavior (*r_s_* = .31, *p* < .001), DSM somatic problems (*r_s_* = .37, *p* .001), DSM conduct problems (*r_s_* = .43, *p* < .001), and post-traumatic stress problems (*r_s_* = .44, *p* < .001) showed a low but clear correlation with C_EFPT. In particular, among behavioral problems, DSM-affective problems (*r_s_* = .54, *p* < .001) and thought problems (*r_s_* = .55, *p* < .001) had a higher correlation with C_EFPT at a moderate level. Among the parental factors, the maternal factor had a higher rank correlation with C_EFPT than the paternal factor. Mother’s depression (*r_s_* = .32, *p* < .001) and parenting stress (*r_s_* = .40, *p* < .001) showed a low but clear correlation with C_EFPT. Home environment factors had a very low correlation with C_EFPT in the range of .07 to .17 compared to other factors (Figure 4).

### 3.3. Performance Evaluation of BNL

We analyzed the performance of all five learning models using the Bayesian network algorithm. The purpose of learning is to classify groups in which EF difficulty remains relatively high, such as in classes 2 or 3, in the entire sample. For example, in the first model (class 1: class 2+3) presented in Table 4, a group in which classes 2 and 3 are mixed is considered a positive sample, and class 1 is considered a negative sample.

Among the five models, the performance of Model 3 (AUC = .97) and Model 4 (AUC = .93) when the threshold is .5 for discriminating class 3, is excellent. Even under the threshold .85 where the model path becomes simpler, Model 3 (AUC = .96) and Model 4 (AUC = .91) are excellent.

**Figure 5:**
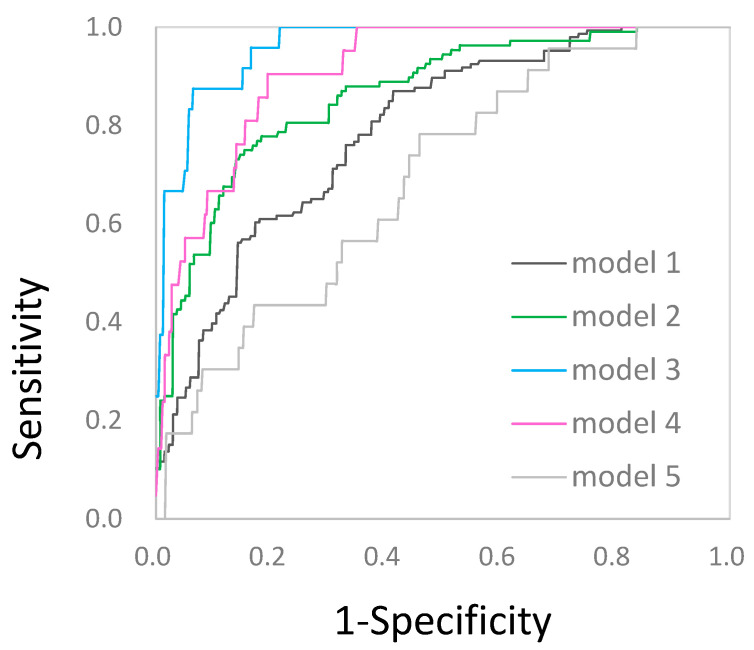
ROC curve. In the Bayesian network approach, the predictive performance (threshold = .85) of Model 3 and 4 are excellent and Model 2 is good.

Compared to the case where the threshold is the average of the arc intensity, the pruning models with the threshold raised to .85 have a much simpler network structure, but the classifier performance is almost the same for Model 3 and Model 4. Therefore, if we are to select a model where the threshold is .85 and examine its performance, the accuracy of Model 4 is 81% (76–86%); its sensitivity is 86% (64–97%), and its specificity is 81% (76–86%). The AUC is .91 (.86-.96). That is, the AUC performance of the model that identifies children whose EFD is in the high class among all children is excellent (Figure 5).

#### 3.3.1. Comparison with xgBoosting

The accuracy of Model 3 using the xgBoost algorithm is 95% (91–98%); the sensitivity is 96% (79–100%); the specificity is 95% (90–98%), and the AUC is .98 (.97–1.00). In addition, the accuracy of Model 4 using the xgBoost algorithm is 92% (89–95%); the sensitivity is 90% (70–99%); the specificity is 93% (89–95%), and the AUC is .98 (.96–1.00). The performances of the models for classifying children in class 3 are excellent (Table 4).

#### 3.3.2. Bayesian Network Structural Analysis and Predictors

**Figure 6:**
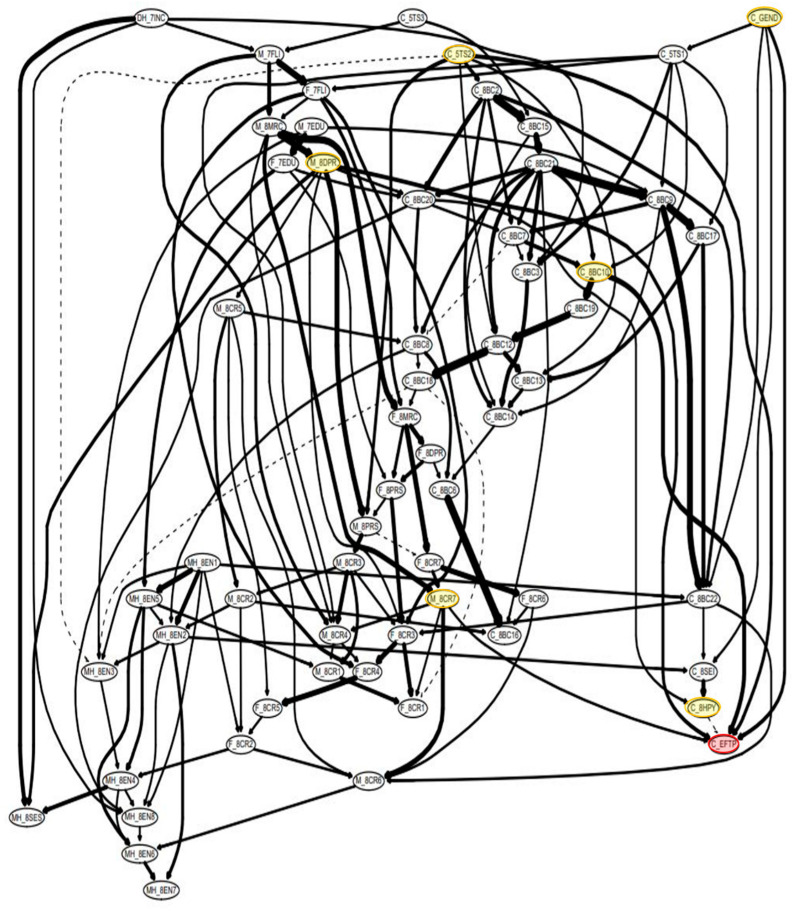
Class 1+2: Class 3 (threshold = .85). Bayesian network structure. Each node is a factor. The final effect node (red) in this study is a label indicating the type of EFD of each learning model (Bootstrapping = 2000). The red circles are the Effect nodes (C_EFPT) of model 4 (threshold = .85) and the yellow circles are the parent nodes (C_GEND, C_5TS2, C_8HPY, C_8BC10, M_8DPR, M_8CR7).

In the five BNL models used in this study, the parent node of the effect node is the final predictor that becomes an input to the linear regression algorithm that predicts the pattern of the child’s EFD longitudinal trajectory. The model using a threshold of .85 has a simpler network and fewer parent nodes than the model using the average threshold. Nevertheless, there is little difference in the predictive performance of Model 4. Therefore, if we analyze the Bayesian network structure to understand the factors that persist in children’s EFD, it is then helpful to examine the structure of Model 4 with a threshold of .85 (Figure 6).

First, in the model 4 network structure, there are 6 parent nodes of the effect node: the child’s gender, the child’s temperamental emotionality, the child’s happiness, the DSM anxiety problems in the first year of elementary school, the mother’s depression, and the coparenting conflict recognized by the mother. In fact, these factors distinguish Class 3 from the other classes. In all five classification models, children’s gender and temperamental emotionality are common predictors. The children’s happiness is a common predictor of Models 3 and 4. The mother’s depression is a common predictor of Models 1 and 2, and the mother’s parenting stress is a common predictor of Models 3 and 4 (Table 4, Figure 6).

The children’s happiness (Models 3 and 4), the mother’s depression (Models 1 and 2), the mother’s parenting stress (Models 3 and 4), and the coparenting conflict recognized by the mother (Models 4 and 5) were common predictors. In addition, various children’s behavioral problems, family factors, and home environments were found to be predictors. These included DSM-affective problems (Model 2); attention problems, thought problems, and DSM conduct problems (Model 3); DSM anxiety problems (Model 5); father’s parenting stress (Model 3); coparenting integrity recognized by the father (Model 5); encouragement of maturity and learning materials at home (Model 3) (Table 4 and Table 5).

Although xgBoosting shows a better predictive performance than Bayesian network modeling, more than 20 predictors are required. On the other hand, Bayesian network modeling shows a good or an excellent performance with relatively few predictors in Models 3 and 4. Further, unlike xgBoosting, Bayesian network modeling structurally shows the relationship between variables. In Model 4, children’s temperamental emotionality and sociality have a cascading effect on children’s behavioral problems as well as family and home environment factors. After these reciprocal influences, the child’s factors (gender, temperamental emotionality, happiness, and DSM anxiety problems) and mother’s factors (depression and coparenting conflict recognized) become final predictors of high EFD longitudinal patterns (Figure 6).

**Figure 7:**
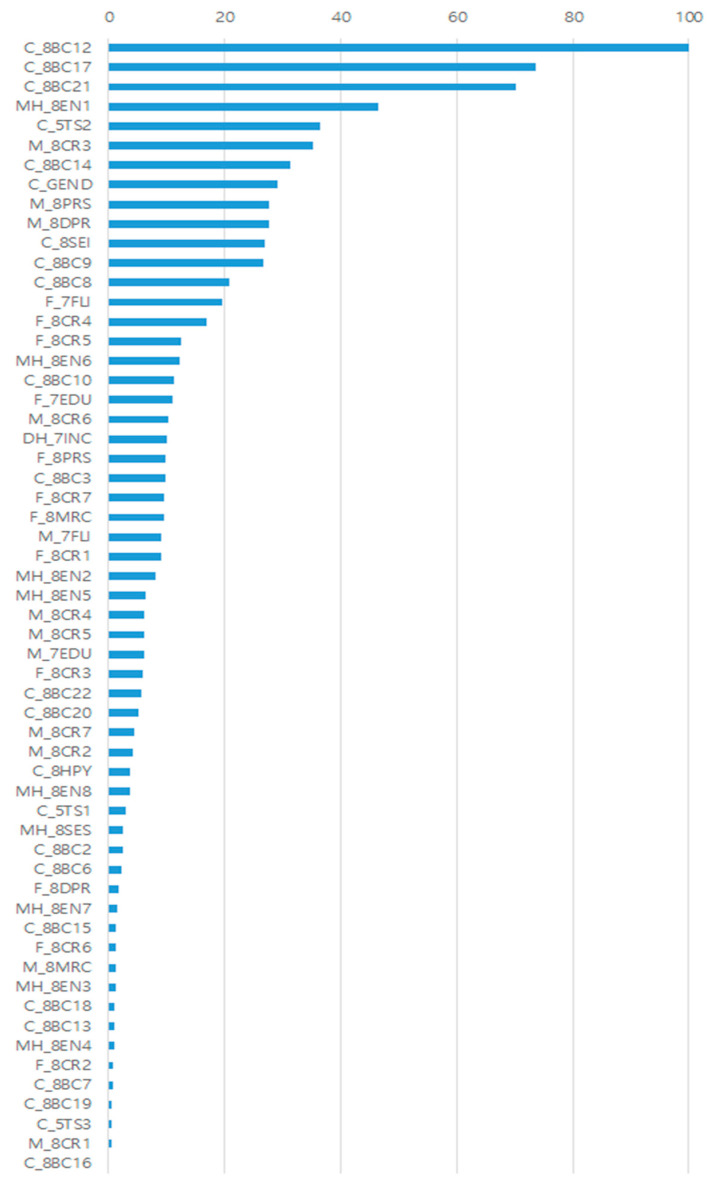
The relative importance of input variables. Assuming that the contribution of the variable with the highest contribution to the prediction is 100, the contribution of the remaining variables is expressed as a relative ratio.

In the xgBoosting modeling of Model 4, the predictors’ contributions are ordered in descending order: DSM attention-deficit/hyper activity problems, thought problems, Posttraumatic stress problems, responsivity at home, temperamental emotionality, warm parenting recognized by the mother, withdrawn/depressed, gender, depression and parenting stress recognized by the mother, etc. Variables related to children’s behavioral problems are presented in order of the highest contribution (Figure 7).

## 4. Discussion

We classified the longitudinal trajectory pattern of EFD using LGCA and panel data until first graders in a Korean elementary school reached the fourth grade (waves 8–11). To identify more vulnerable patterns among the classified children’s EFD trajectory patterns, the performance of modeling with predictors (waves 5–8) was compared using BNL and xgBoosting.

We hypothesized that the combination of caregivers and children’s emotional problems related to the child’s early ability to regulate will later increase the severity of the child’s EFD. In the first year of life, lower regulatory capacity in infants and higher levels of maternal depression were predictive of depression-like symptom of the toddler (Gartstein and Bateman 2008). Negative experiences of being devalued in interpersonal relationships can shape thought problems (Kools 1997). Likewise, difficulties in regulatory capacity early in life are likely to lead to negative emotional experiences and thought problems. Meanwhile, parenting stress predicted future parental depression (Thomason et al. 2014). The emotional problems of children and parents that form during early parenting can lead to a chain of other mental problems. Consequently, the severity of the child’s EFD will persist as the child’s and caregiver’s problems are combined.

### 4.1. The Usefulness of LCGA to Analyze EFD Longitudinal Trajectory Patterns

As a result of performing LGCA, there was a class in which the longitudinal trajectory of EFD (waves 8–11) was maintained at a low level; the class was maintained at a high level and at the intermediate level between the two classes. The difference in EFD among the three groups over four years was statistically significant. This result supports the existence of individual differences in EF development with different trajectories (Friedman et al. 2011).

In this study, the longitudinal trajectory of EFD in elementary school children increased gradually on average from the first to the third grade and then decreased in the fourth grade. The group with the highest level of EFD had a greater decrease in EFD during grades 3 and 4. However, these results are contrary to a study comparing the EF of children in the first and sixth grades of elementary school, which showed that the higher the grade, the more significant the improvement in EF (Lee and Hong 2006). In this study, children’s EFD increased slowly and steadily after entering primary school. This was similar to a report that found EF improved rapidly in preschoolers but developed at a slower pace after entering elementary school (Best and Miller 2010; De Luca and Leventer 2008).

However, the continued increase in children’s EFD risk after entering elementary school may reflect difficulties in adjusting to school. In previous studies, EFD was the most important contributing variable in predicting school adaptation of Korean elementary school students (Goh and Jeon 2020). In Korea, there is a cultural difference between early childhood education, in which an integrated and flexible curriculum is operated, and school-age education, in which learning evaluation between students is important. Children in the group with the highest EFD levels may have had more difficulty performing goal-oriented behavior in these elementary school cultures, and it would not have been until the fourth grade that the difficulty was reduced (Goh and Jeon 2020).

In addition, Korea’s high educational expectations are related to psychological risks, including stress, depression, anxiety, and even suicide, among children (Phosaly et al. 2019). Children’s internalizing and externalizing of behavioral problems (Wang and Zhou 2019; Tillman et al. 2015) were longitudinally associated with EFD. Therefore, it is necessary to select and support children who experience more behavioral problems and EFDs due to changes in educational culture. The LCGA is a useful methodology for selectively classifying children with persistent EFDs, and it is being used as a basis for preparing future support.

### 4.2. The Usefulness of BNL to Analyze Predictors of EFD Longitudinal Trajectories

Analyzing the network structure using BNL helps identify the main predictors of EFD during elementary school (waves 8–11) from previous data (waves 5–8) and understand the relationship among the different factors.

First, in the model that identifies children with high EFD longitudinal patterns, the main predictors were the child’s gender, temperamental emotionality, happiness, DSM anxiety problems, the mother’s depression, and coparenting conflict recognized by the mother. In the Bayesian network structure, the child’s gender and negative emotional temperament directly becomes the parent node of the effect node. In particular, children’s temperamental emotions are important in that they are connected to anxious/depressed, rule-breaking behavior, post-traumatic stress problems, aggressive behaviors, and DSM anxiety problems, and these behavioral problems predict a high level of EFD. Meanwhile, the main predictors of other modeling were children’s gender, negative emotional temperament, affective-related problems, and the mother’s parenting stress, which had a relationship to marital conflict and depression.

Whereas, hot EFs are related to brain functions that control emotional or motivational responses to stimuli, cold EFs are related to functions of control in emotionally neutral contexts (Zelazo and Carlson 2012). In particular, the hot EF is responsible for gratification delay ability, emotion regulation, and emotional decision-making functions (Bernabei et al. 2018). Our results are similar to previous studies showing that hot EF in early childhood is associated with emotional problems (McIntyre et al. 2006; Poon 2018). In addition, these results are similar to reports that show that hot EF development predicts cognitive and self-regulating abilities in adolescence (Mischel et al. 1988; Shoda et al. 1990), and the lack of cool EF in young children can be an indicator of a serious behavior problem (Hughes et al. 2000; Riggs et al. 2003; Poon and Ho 2014).

Meanwhile, the mother’s depression and parenting stress also become the final parent node of the effect node through the child’s temperament and marital conflict. The results of this study support the reports that the quality of the marital relationship predicts a mother’s depression (Faisal-Cury et al. 2020; Thomason et al. 2014; Park et al. 2018; Power et al. 2021), and parenting stress (De Cock et al. 2017) negatively affects children’s EF.

In all of our discriminative models, gender differences and children’s temperament were common predictors (parent nodes) of the effect node in identifying risk groups. These findings are similar to a report that suggests that before school age, girls may be more advantageous in inhibition than boys (Ardila et al. 2005; Klenberg et al. 2001). However, it is different from the report in that there is no difference according to gender after school age (Jacobsen et al. 2017; Park and Lee 2013). Even in elementary school, the EFD development patterns of Korean children were still related to gender differences.

In this study, temperamental emotionality was also a direct predictor (parent node) of the effect node, similar to the report that temperamental difficulties are related to cognitive EF and aggressive and antisocial behavioral problems (Giancola et al. 1998). In addition, if we trace the antecedent conditions of the final predictors in the two discrimination models of this study, they have a commonality in that the child’s temperament and the parent’s family interaction are linked in a directional structure. These results are similar to reports that children’s EF development is related to harmonious compatibility between children’s temperament and parental parenting in early life (Lee and Kim 2020). In addition, our two Bayesian network models showed a structure in which a child’s temperament has a pathway for predicting their various behavioral problems and a pathway for predicting maternal depression as well as parenting stress through family interactions. These results support the important influence of children’s temperament emotionality (Leve et al. 2013) and family interactions (Hughes et al. 2020; Ponnet et al. 2013) on EF development and support an approach that considers both genetic and environmental factors in researching EF development (Zelazo 2013).

In our study, children’s happiness and home environment factors were also major predictors. It has been reported that positive psychological factors, such as children’s self-esteem (Bajaj et al. 2016; He et al. 2021) and happiness (Choi and Choi 2019; Sung and Choi 2021) also affect the development of children’s EF. In addition, a rich home environment is favorable to children’s EF (Dilworth-Bart et al. 2007; Sarsour et al. 2011). Therefore, at least in the case of Korean children, children with temperamental difficulties in early life may need special attention and preventive support for the developmental trajectory of EFD in elementary school.

Second, using BNL helps identify children at risk of EFD with a good predictive performance during the elementary school years (waves 8–11). Using BNL, the performance of modeling to classify higher EFD levels was excellent (Model 4: AUC = .91). The results of this study show that LCGA and BNL are very useful for classifying the EFD longitudinal trajectory pattern during the elementary school period (waves 8–11) by inputting data from the previous period (waves 5–8).

In this study, the performance of the xgBoosting models was better than that of the BNL models. In particular, the performance of children belonging to Class 3 that need the most support is excellent (Model 4 AUC = .98). BNL shows a good predictive performance and has the advantage of visually showing the causal structure between nodes.

### 4.3. Limitations and Suggestions

First, the EFD in this study has limitations because it uses only data from Korean children and their mothers. In the PSKC data, there are data evaluated by mothers and teachers in the first grade of elementary school. However, for children in the second grade, only data evaluated by the mother are available. In this study, only the trajectory of the EFD evaluated by the mother was analyzed. In future research, it will be necessary to compare whether the trajectory and predictors of EFD differ from the results of this study by using data evaluated by children and teachers from various cultural backgrounds. In addition, in this study, the EFD data were collected by self-report measurements, which constitute a limitation. For a more objective analysis, it is necessary to secure consistent data through direct measurement of EF.

Even considering these limitations, this tool was useful in predicting the difficulties of EFs in real contexts. When the evaluation tools for EFs are classified into performance-based and rating measures (Toplak et al. 2013), Song’s (2014) tool corresponds to the rating measure. This tool focuses on the ecological validity in assessing whether children have goal-directed control in real life. In our results, the EFD of children classified into the three groups remained at a longitudinally stable level. Parents consistently assessed their children, and these assessments were useful for the studies predicting EFD in children’s real life.

Second, in this study, the developmental trajectory of EFD was established through LCGA. According to our interpretation of this, the developmental trajectory of EFD tends to be maintained consistently by level. In a follow-up study, it is necessary to reveal how EFD transitions over time and what factors affect the transition through latent transition analysis. Variables that predict or correlate with EF or EFD identified in previous studies have been reported in various areas: child-related (Jacobsen et al. 2017; Sung and Choi 2021; Tillman et al. 2015; Wang and Zhou 2019), parent-related (Altenburger and Schoppe-Sullivan 2021; De Cock et al. 2017; Gustafsson et al. 2015; Hughes et al. 2013; Lucassen et al. 2015; Meuwissen and Englund 2016), and home-related (Last et al. 2018; Sarsour et al. 2011).

However, this study focused on children at a high risk for longitudinal EFD using a person-centered approach. This is differentiated from previous studies in that the network approach selected variables that predicted these dangerous states to be higher. The network approach can be used clinically in intervention programs to predict or prevent various risk factors belonging to the field of developmental psychopathology, including EFD.

## 5. Conclusions

Utilizing LCGA and BNL is useful for identifying latent layers of EFD longitudinal trajectories and extracting key predictors. In particular, it shows that predictors can identify differences according to the level of EFD risk with a high performance. In addition, the relationship among the predictive factors can be modeled using BNL. Compared to the xgBoosting classifier, the strength of the BNL approach lies in its causal structure, showing the relationship between specific factors and better performance.

In the future, it will be necessary to study whether the EF of children at risk is effectively changed when the intervention is conducted, focusing on the major predictors derived from this study. In addition, since there are various methods of measuring EF, it is necessary to check whether results, such as those in this study, will be obtained even for the developmental changes in EF measured by other methods.

## Figures and Tables

**Table 1:** Variables measured over time.

Time	Predictor Classification	Measured Variable
5th (3-year-olds)	child-related predictors	C_5TS1 = Activity as a subvariable of EAS; C_5TS2 = Emotionality as a subvariable of EAS; C_5TS3 = Sociality as a subvariable of EAS
7th (5-year-olds)	parent-related predictors	M_7FLI = Family interaction recognized by the mother; F_7FLI = Family interaction recognized by the father; M_7EDU = Educational level of the mother; F_7EDU = Educational level of the mother
	home-related predictors	DH_7INC = Monthly income
8th (6-year-olds)	child-related predictors	C_8SEI = Self-esteem; C_8HPY = Happiness; C_8BC2 = Anxious/depressed; C_8BC3 = Somatic complaints; C_8BC6 = Attention problems; C_8BC7 = Aggressive behaviors; C_8BC8 = Other problems; C_8BC9 = DSM-affective problems; C_8BC10 = DSM anxiety problems; C_8BC12 = DSM attention-deficit/hyper activity problems; C_8BC13 = DSM oppositional defiant problems; C_8BC14 = Withdrawn/depressed; C_8BC15 = Rule-breaking behavior; C_8BC16 = Social problems; C_8BC17 = Thought problems; C_8BC18 = DSM somatic problems; C_8BC19 = DSM conduct problems; C_8BC20 = Obsessive–compulsive symptom; C_8BC21 = Post-traumatic stress problems; C_8BC22 = Sluggish cognitive tempo.
	Parent-related predictors	M_8DPR = Depression recognized by the mother; M_8PRS = Parenting stress recognized by the mother; M_8MRC = Marital conflict recognized by the mother; M_8CR1 = Parent–child interaction of mother; M_8CR2 = Controlled parenting of mother; M_8CR3 = Warm parenting recognized by the mother; M_8CR4 = Coparenting integrity recognized by the mother; M_8CR5 = Coparenting reprimand recognized by the mother; M_8CR6 = Coparenting disparagement recognized by the mother; M_8CR7 = Coparenting conflict recognized by the mother; F_8DPR = Depression recognized by the father; F_8PRS = Parenting stress recognized by the father; F_8MRC = Marital conflict recognized by the father; F_8CR1 = Parent–child interaction recognized by the father; F_8CR2 = Controlled parenting recognized by the father; F_8CR3 = Warm parenting recognized by the father; F_8CR4 = Coparenting integrity recognized by the father; F_8CR5 = Coparenting reprimand recognized by the father; F_8CR6 = Coparenting disparagement recognized by the father; F_8CR7 = Coparenting conflict recognized by the father
	home-related predictors	MH_8SES = Subjective SES; MH_8EN1 = Responsivity; MH_8EN2 = Encouragement of maturity; MH_8EN3 = Emotional climate; MH_8EN4 = Learning materials and opportunities; MH_8EN5 = Enrichment; MH_8EN6 = Family companionship; MH_8EN7 = Family integration; MH_8EN8 = Physical environment.
	Response variable	EFDs = executive function difficulties
9th (7-year-olds)	response variable	EFDs = executive function difficulties
10th (8-year-olds)	response variable	EFDs = executive function difficulties
11th (9-year-olds)	response variable	EFDs = executive function difficulties

**Table 2:** Latent Class Growth model for EFD longitudinal pattern in children.

Number of Classes	loglik	AIC	BIC	SABIC	Entropy	Class 1 (%)	Class 2 (%)	Class 3 (%)	Class 4 (%)
1	−1059.60	2129.20	2153.77	2137.89	1.00	100.00			
2	−186.55	393.10	442.24	410.48	.86	65.21	34.79		
3	236.97	−443.94	−370.23	−417.87	.89	50.69	40.76	8.65	
4	338.75	−637.51	−539.23	−602.75	.83	42.45	32.01	19.68	5.86

The most suitable model with low log-likelihood, AIC, BIC, and SABIC values and high entropy values is the model with three latent layers.

**Table 3:** Differences among latent layers of children’s EFD at each wave (time).

Wave	Total (*n* = 1006)	Class 1 (*n* = 509)	Class 2 (*n* = 410)	Class 3 (*n* = 87)	*F*	*p*
	m ± sd	m ± sd	m ± sd	m ± sd		
w8	1.44 ± 0.30	1.22 ± 0.15	1.59 ± 0.21	1.97 ± 0.27	796.83	<.001
w9	1.47 ± 0.31	1.24 ± 0.14	1.63 ± 0.19	2.04 ± 0.23	1129.95	<.001
w10	1.48 ± 0.33	1.25 ± 0.16	1.62 ± 0.19	2.14 ± 0.26	1091.39	<.001
w11	1.43 ± 0.32	1.22 ± 0.16	1.57 ± 0.21	2.07 ± 0.23	906.85	<.001

As the one-way ANOVA shows, there was a significant difference in the EFD mean among the three latent layers (class 1 < class 2 < class 3, *p* < .001).

**Table 4:** Predictive performance of children’s EFD modeling.

	Model	0:1	Threshold	Accuracy	Sensitivity	Specificity	AUC
Bayesian network modeling	1	class 1: class 2+3	.50	.80 (.75–.84)	.86 (.79–.91)	.85 (.78–.91)	.86 (.82–.90)
	2	class 1: class 2	.50	.79 (.73–.84)	.74 (.65–.82)	.83 (.76–.89)	.87 (.82–.91)
	3	class 1: class 3	.50	.92 (.87–.96)	.96 (.79–1.00)	.91 (.85–.95)	.97 (.95–.99)
	4	class 1+2: class 3	.50	.82 (.77–.87)	.86 (.64–.97)	.82 (.77–.87)	.93 (.90–.97)
	5	class 2: class 3	.50	.72 (.64–.80)	.78 (.56–.93)	.71 (.62–.79)	.84 (.77–.92)
	1	class 1: class 2+3	.85	.69 (.63–.74)	.68 (.60–.76)	.69 (.61–.77)	.79 (.74–.84)
	2	class 1: class 2	.85	.80 (.75–.85)	.74 (.65–.82)	.85 (.78–.91)	.86 (.82–.91)
	3	class 1: class 3	.85	.88 (.82–.92)	.88 (.68–.97)	.88 (.81–.93)	.96 (.93–.99)
	4	class 1+2: class 3	.85	.81 (.76–.86)	.86 (.64–.97)	.81 (.76–.86)	.91 (.86–.96)
	5	class 2: class 3	.85	.59 (.50–.67)	.70 (.47–.87)	.57 (.47–.66)	.69 (.58–.80)
xgBoosting	1	class 1: class 2+3		.87 (.83–.91)	.91 (.85–.95)	.83 (.75–.89)	.96 (.93–.98)
	2	class 1: class 2		.90 (.86–.94)	.94 (.88–.98)	.87 (.80–.92)	.96 (.93–.98)
	3	class 1: class 3		.95 (.91–.98)	.96 (.79–1.00)	.95 (.90–.98)	.98 (.97–1.00)
	4	class 1+2: class 3		.92 (.89–.95)	.90 (.70–.99)	.93 (.89–.95)	.98 (.96–1.00)
	5	class 2: class 3		.93 (.87–.96)	.87 (.66–.97)	.94 (.87–.99)	.95 (.87–1.00)

Output variables (C_EFPT) are classified as 0 or 1. For example, class 1 and class 2 have a value of 0 and class 3 has a value of 1 in model 4. In order to analyze the network structure, it is good to consider the thresholds related to the arc intensity distribution. If the threshold is taken as the average value of the arc intensity, the network is quite dense, so it is not easy to analyze the structure. However, if the threshold is raised to the .85 level, the network structure becomes much simpler and easier to analyze through pruning.

**Table 5:** Predictors of EFD modeling by Bayesian network learning.

Model	0 : 1	Threshold	Effect Node (C_EFPT)’s Parent Node
1	class 1: class 2+3	.50	C_GEND, C_5TS2, C_8BC9, C_8BC17, M_8PRS
2	class 1: class 2	.50	C_GEND, C_5TS2, C_8HPY, C_8BC9, M_8PRS, MH_8EN2
3	class 1: class 3	.50	C_GEND, C_5TS2, C_8SEI, C_8HPY, C_8BC6, C_8BC9, C_8BC10, C_8BC17, C_8BC19, C_8BC20, M_8DPR, M_8CR5, M_8CR7, F_8PRS, F_8CR4, F_8CR6, MH_8SES, MH_8EN2, MH_8EN4, MH_8EN8
4	class 1+2: class 3	.50	C_GEND, C_5TS2, C_8HPY, C_8BC6, C_8BC9, C_8BC10, C_8BC16, C_8BC22, M_8DPR, M_8CR7, F_8CR3, F_8CR4, MH_8EN2, MH_8EN4
5	class 2: class 3	.50	C_GEND, C_5TS2, C_8BC9, C_8BC10, M_8DPR, M_8CR5, M_8CR7, F_7FLI, F_8CR4, MH_8SES
1	class 1: class 2+3	.85	C_GEND, C_5TS2, M_8PRS
2	class 1: class 2	.85	C_GEND, C_5TS2, C_8BC9, M_8PRS
3	class 1: class 3	.85	C_GEND, C_5TS2, C_8HPY, C_8BC6, C_8BC17, C_8BC19, M_8DPR, F_8PRS, MH_8EN2, MH_8EN4
4	class 1+2: class 3	.85	C_GEND, C_5TS2, C_8HPY, C_8BC10, M_8DPR, M_8CR7
5	class 2: class 3	.85	C_GEND, C_5TS2, M_8CR7, F_8CR4

Output variables (C_EFPT) are classified as 0 or 1. For example, class 1 and class 2 have a value of 0 and class 3 has a value of 1 in model 4.

## Data Availability

The datasets generated and/or analyzed during the current study are available in the Panel Study on Korean Children repository, which is available online at https://panel.kicce.re.kr/pskc/index.do (accessed on 9 May 2020).

## Supplementary Materials

The following supporting information can be downloaded at: https://www.mdpi.com/article/10.3390/jintelligence10040074/s1, Figures S1–S5: DAG of bnlearning model 1–5. Figures S6–S10: Distribution of strengths in bnlearning model 1–5. Figures S11–S15: Bayesian network structure in model 1–5 (threshold = .50). Figures S16–S20: Bayesian network structure in model 1–5 (threshold = .75). Figures S21–S25: Bayesian network structure in model 1–5 (threshold = .85). Figures S26–S30: Predictor in xgBoosting model 1–5. Supplementary File S1. Bnlearning and xgBoosting script.
