# Supplementary figures and images for: Application of a Bayesian Network Learning Model to Predict Longitudinal Trajectories of Executive Function Difficulties in Elementary School Students
Source: J Intell. 2022 Sep 23;10(4):74. doi: 10.3390/jintelligence10040074 (PMC9589973; doi:10.3390/jintelligence10040074)

averaged DAG

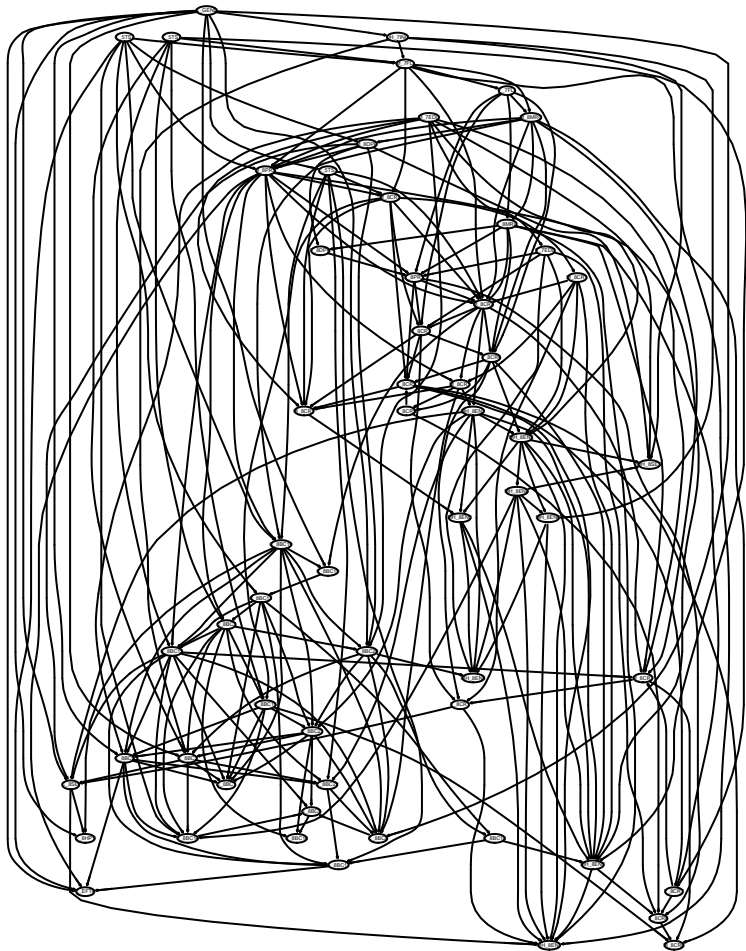

single DAG

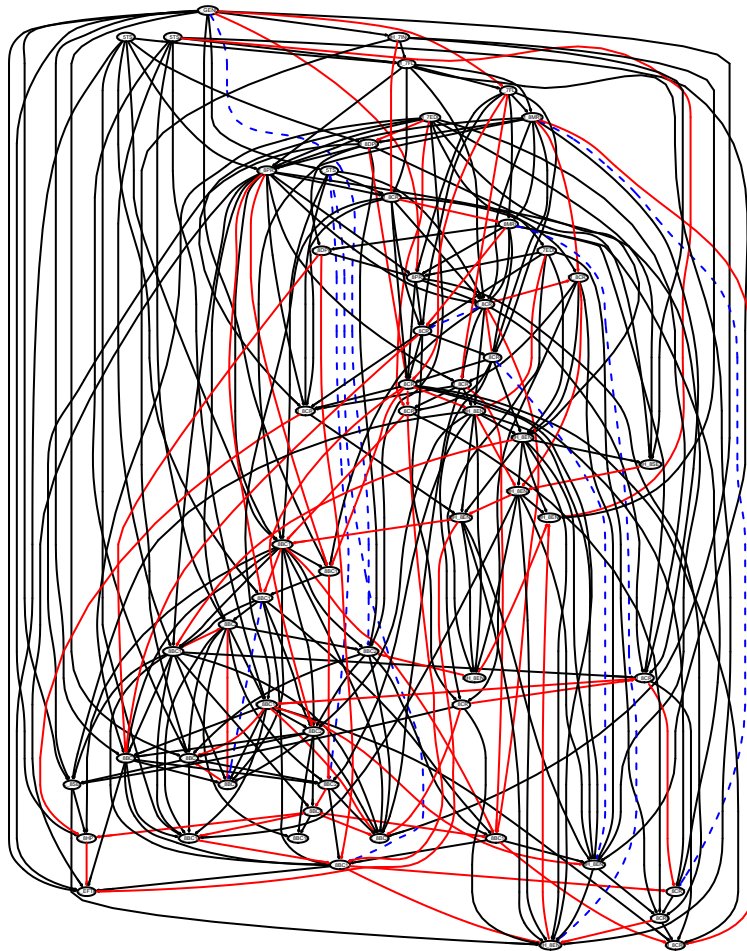

Supplement: Supplementary file 1 [file jintelligence-10-00074-s001.zip › Figure S1 DAG of bnlearning model 1.pdf]

**threshold = 0.5**

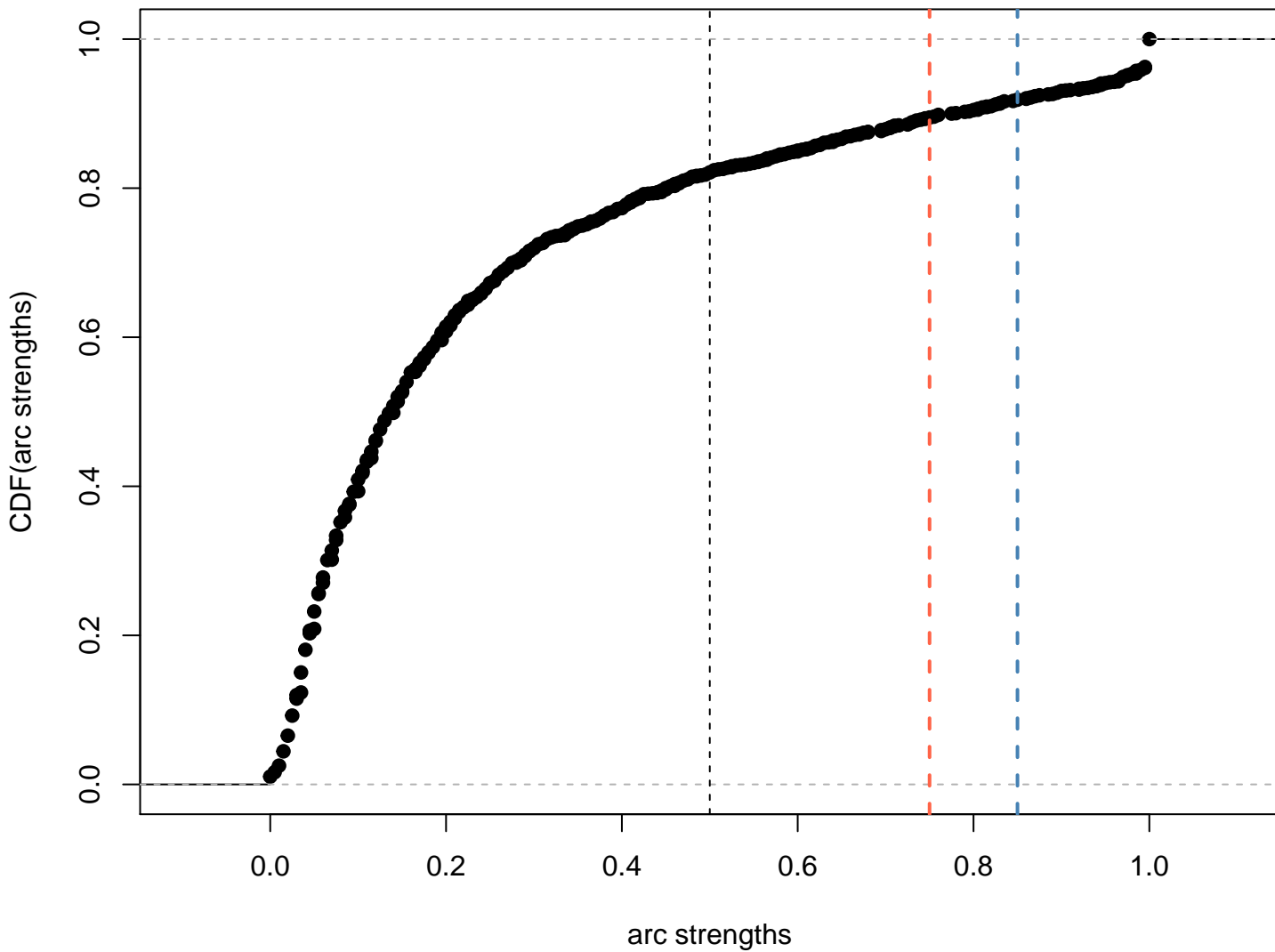

Supplement: Supplementary file 1 [file jintelligence-10-00074-s001.zip › Figure S10 Distribution of arc strengths in bnlearning model 5.pdf]

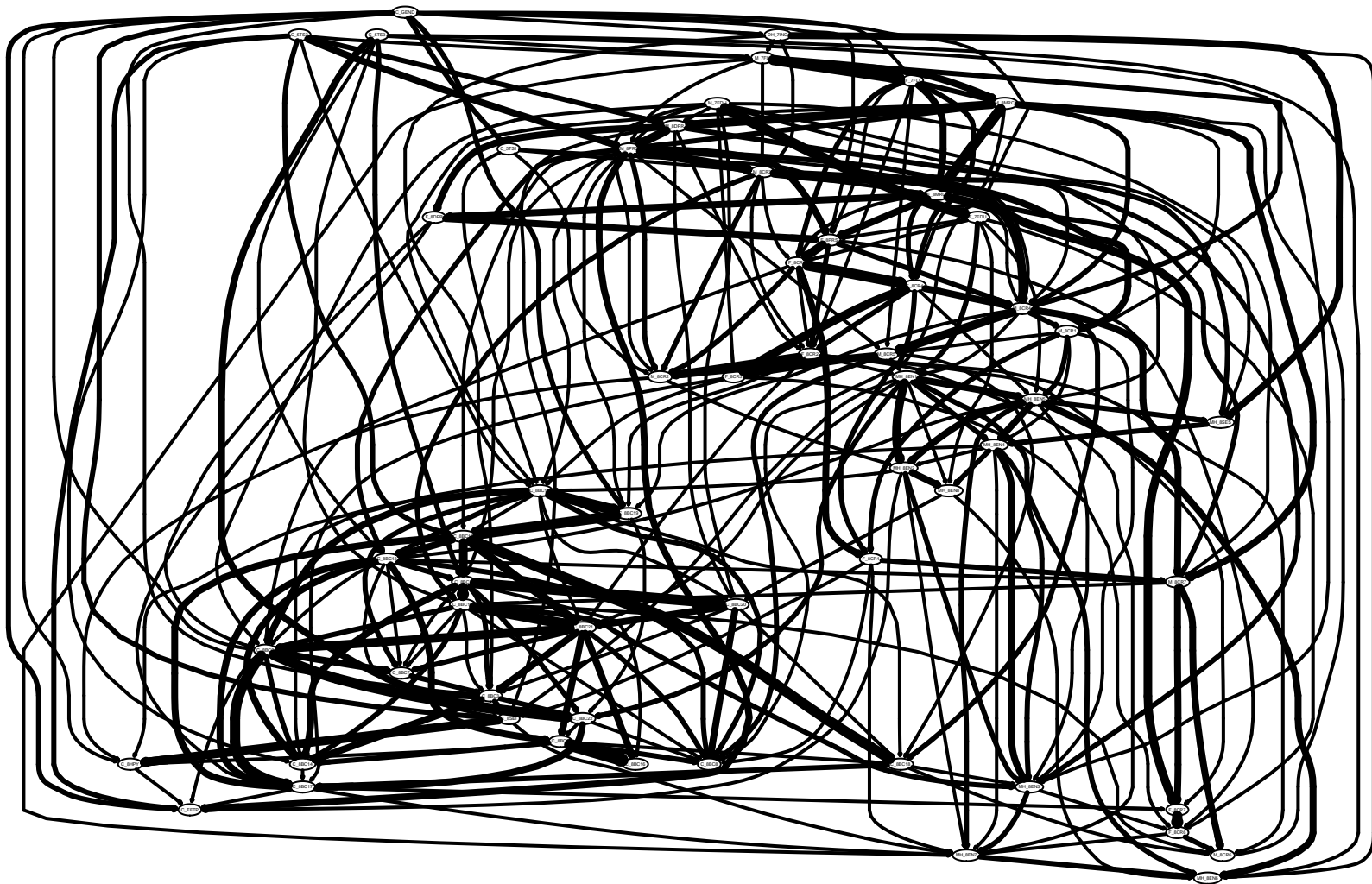

Supplement: Supplementary file 1 [file jintelligence-10-00074-s001.zip › Figure S11 Bayesian network structure in model 1 (threshold=.50).pdf]

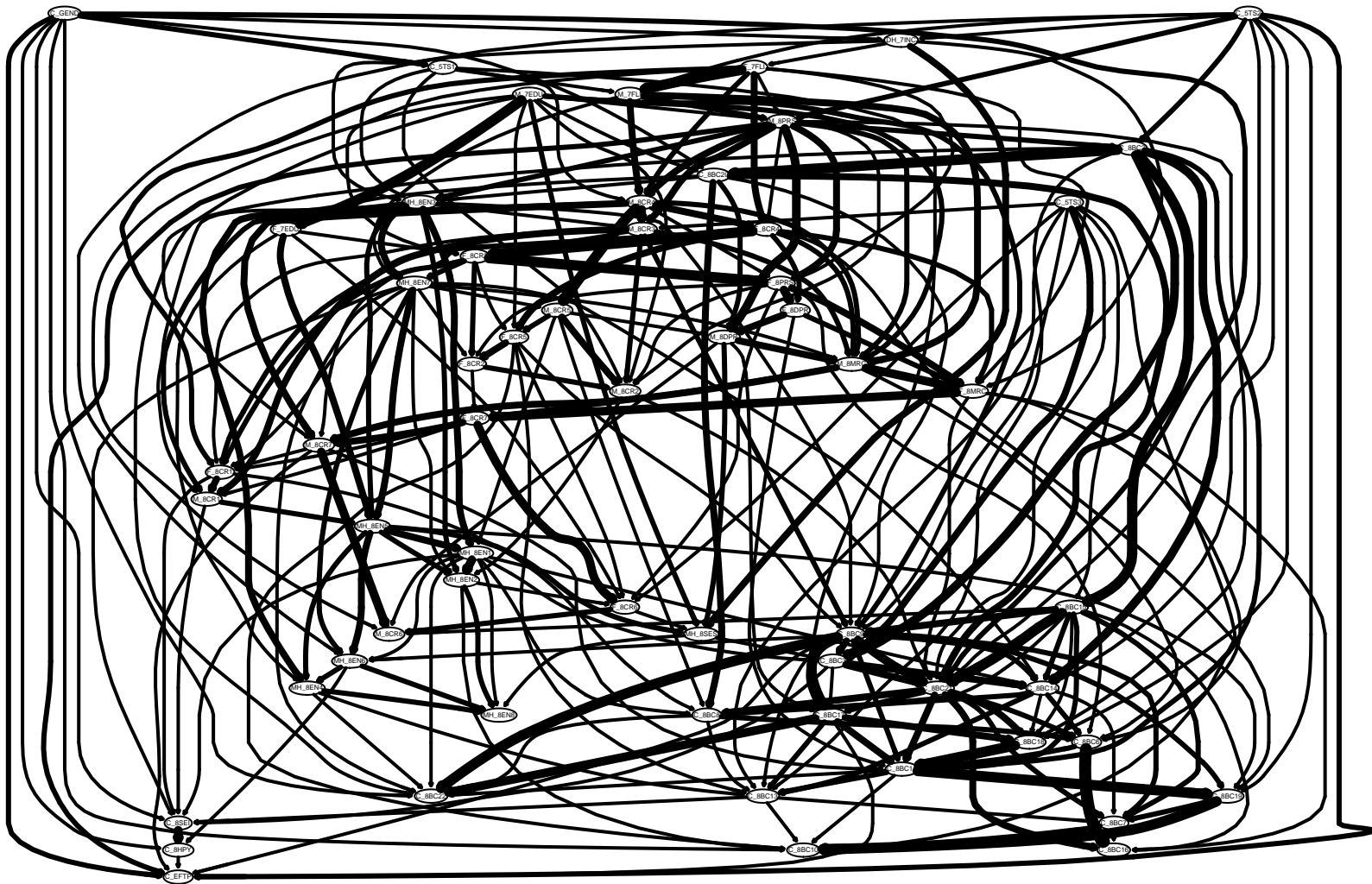

Supplement: Supplementary file 1 [file jintelligence-10-00074-s001.zip › Figure S12 Bayesian network structure in model 2 (threshold=.50).pdf]

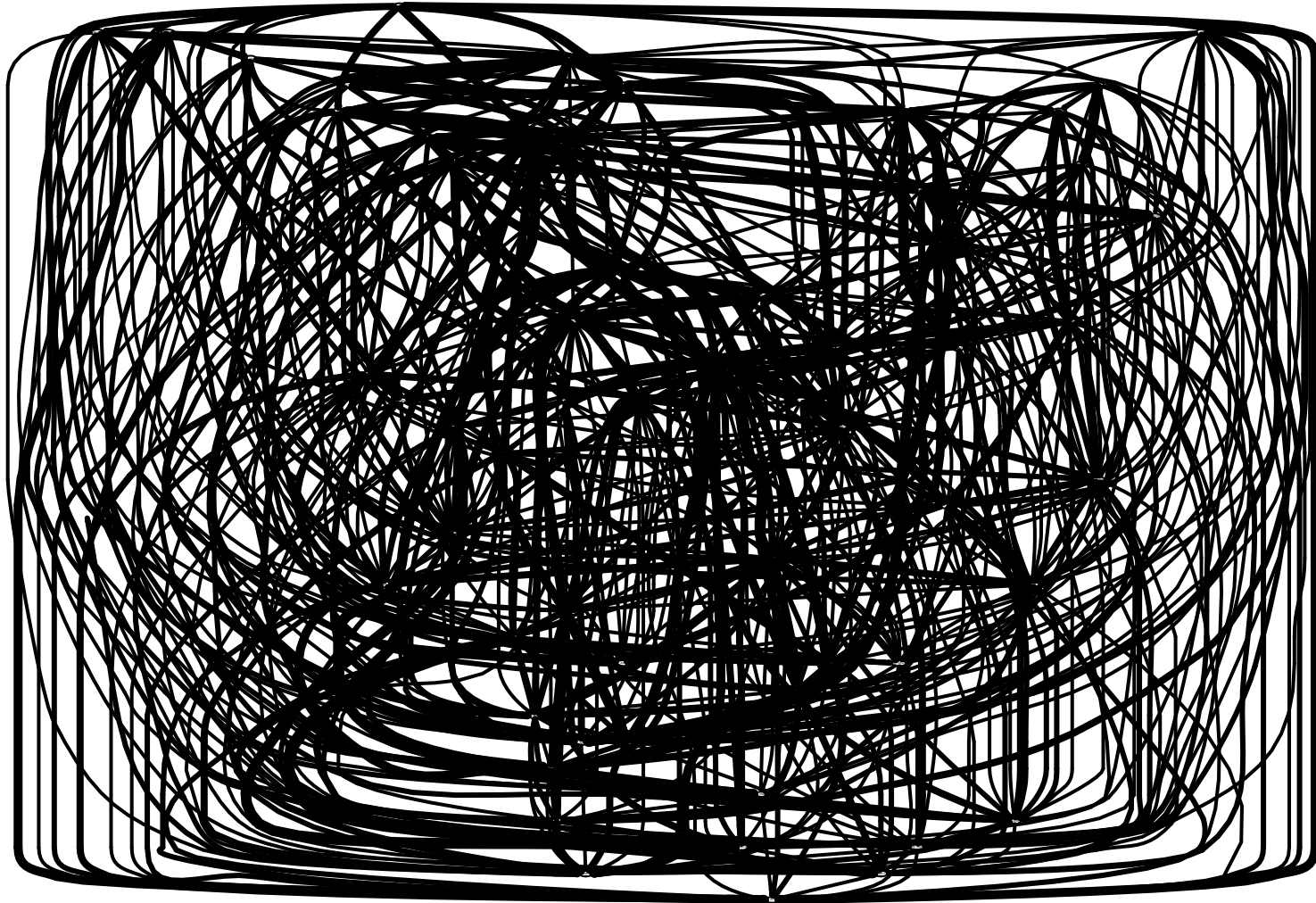

Supplement: Supplementary file 1 [file jintelligence-10-00074-s001.zip › Figure S13 Bayesian network structure in model 3 (threshold=.50).pdf]

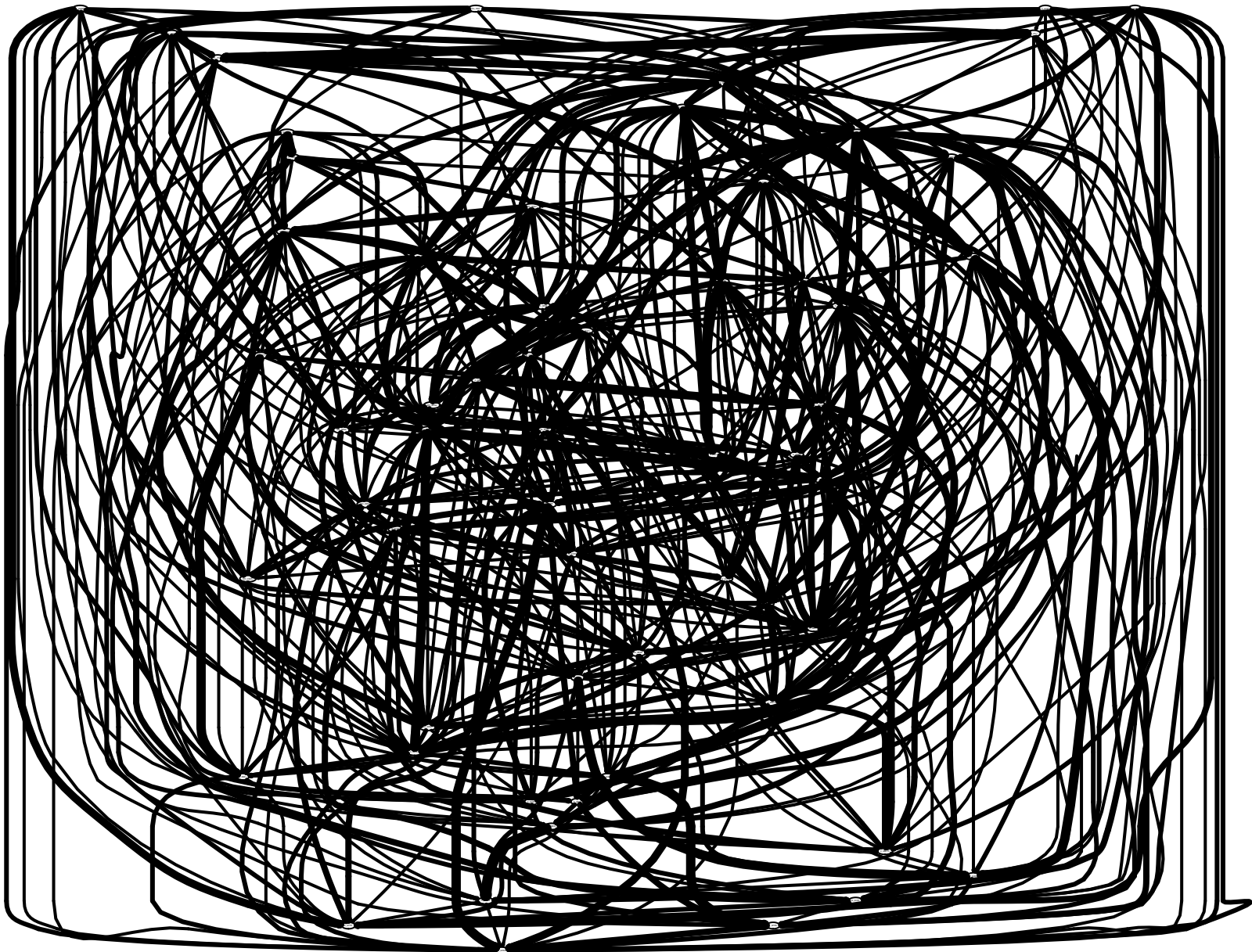

Supplement: Supplementary file 1 [file jintelligence-10-00074-s001.zip › Figure S14 Bayesian network structure in model 4 (threshold=.50).pdf]

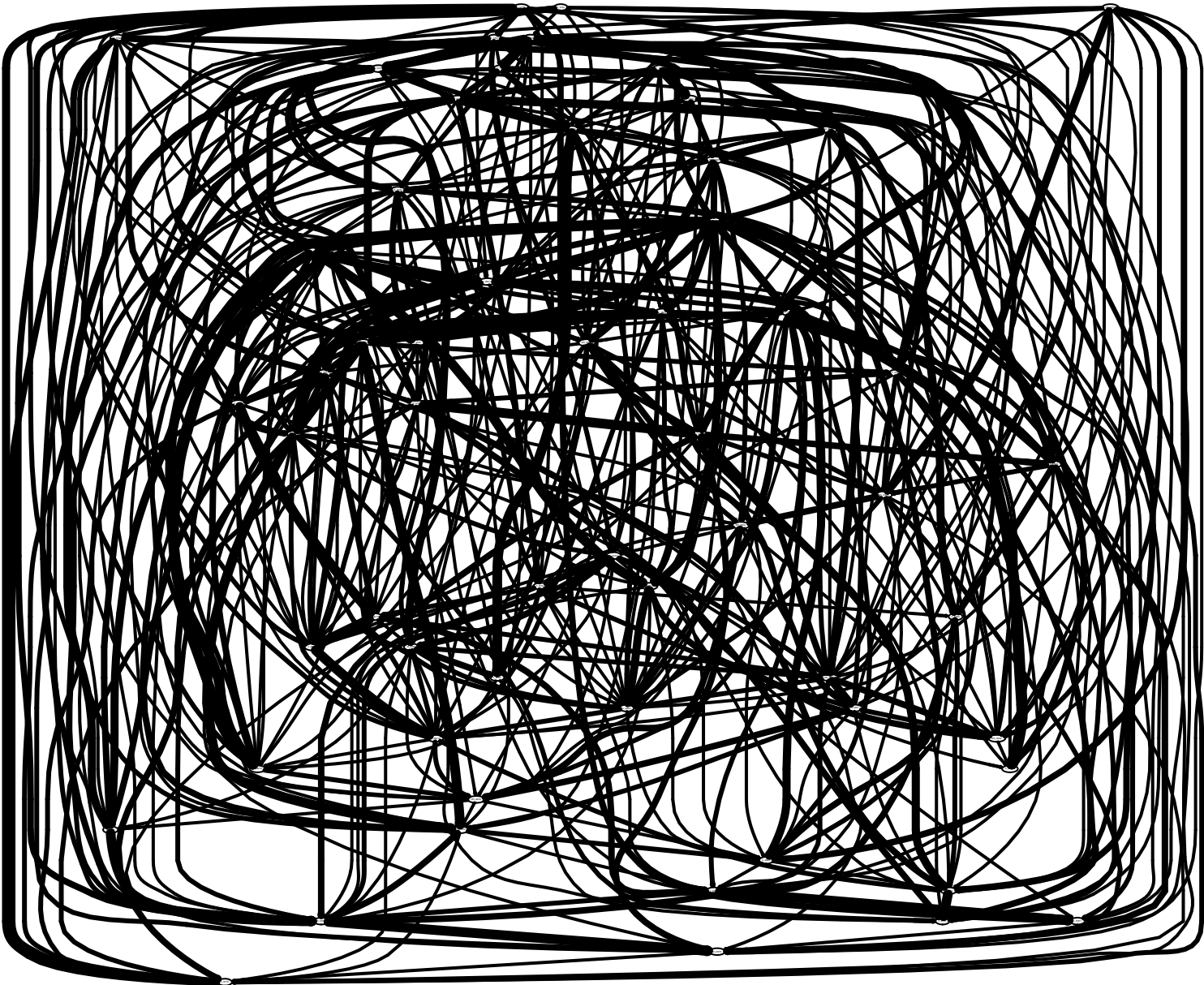

Supplement: Supplementary file 1 [file jintelligence-10-00074-s001.zip › Figure S15 Bayesian network structure in model 5 (threshold=.50).pdf]

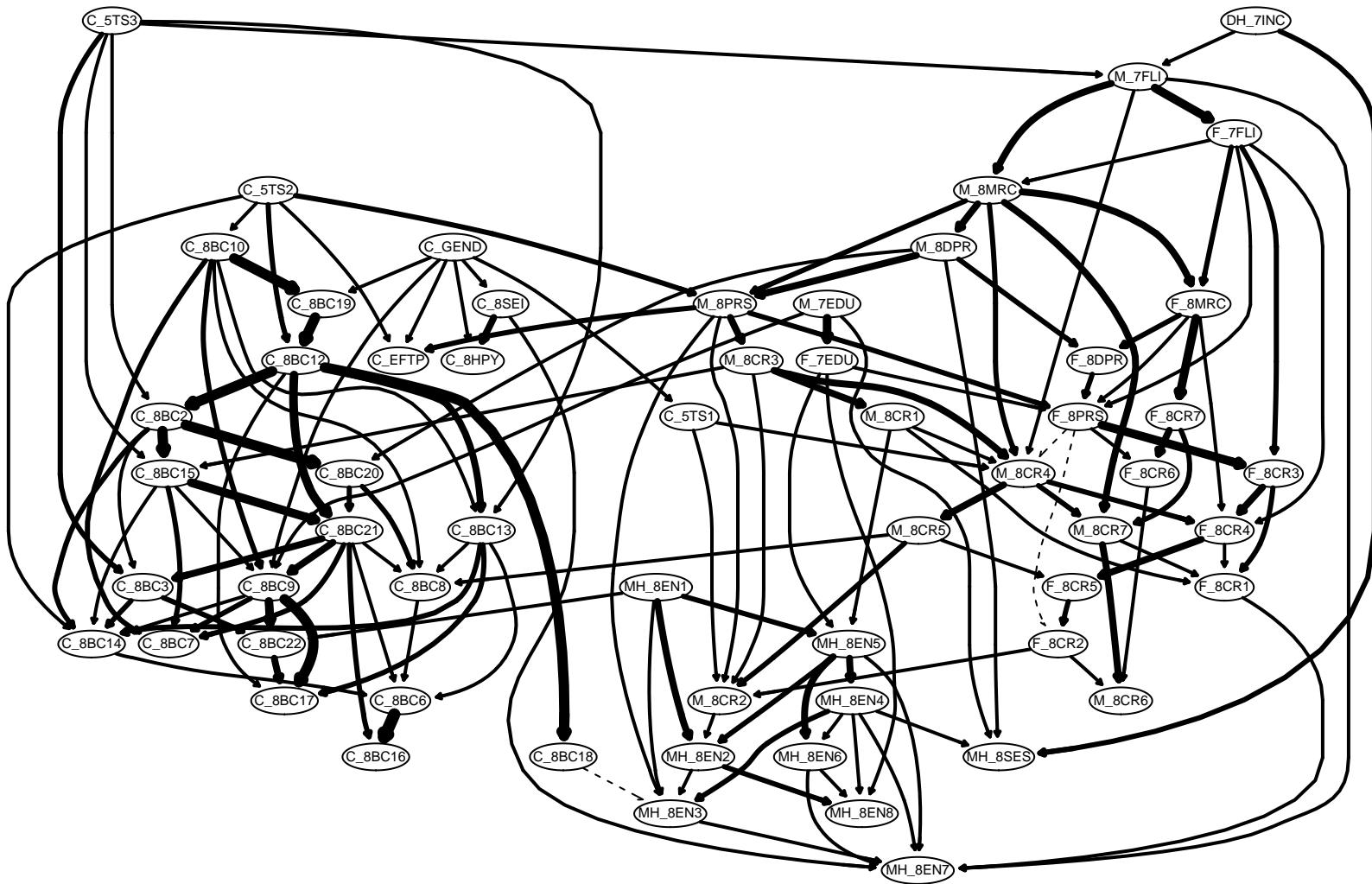

Supplement: Supplementary file 1 [file jintelligence-10-00074-s001.zip › Figure S16 Bayesian network structure in model 1 (threshold=.75).pdf]

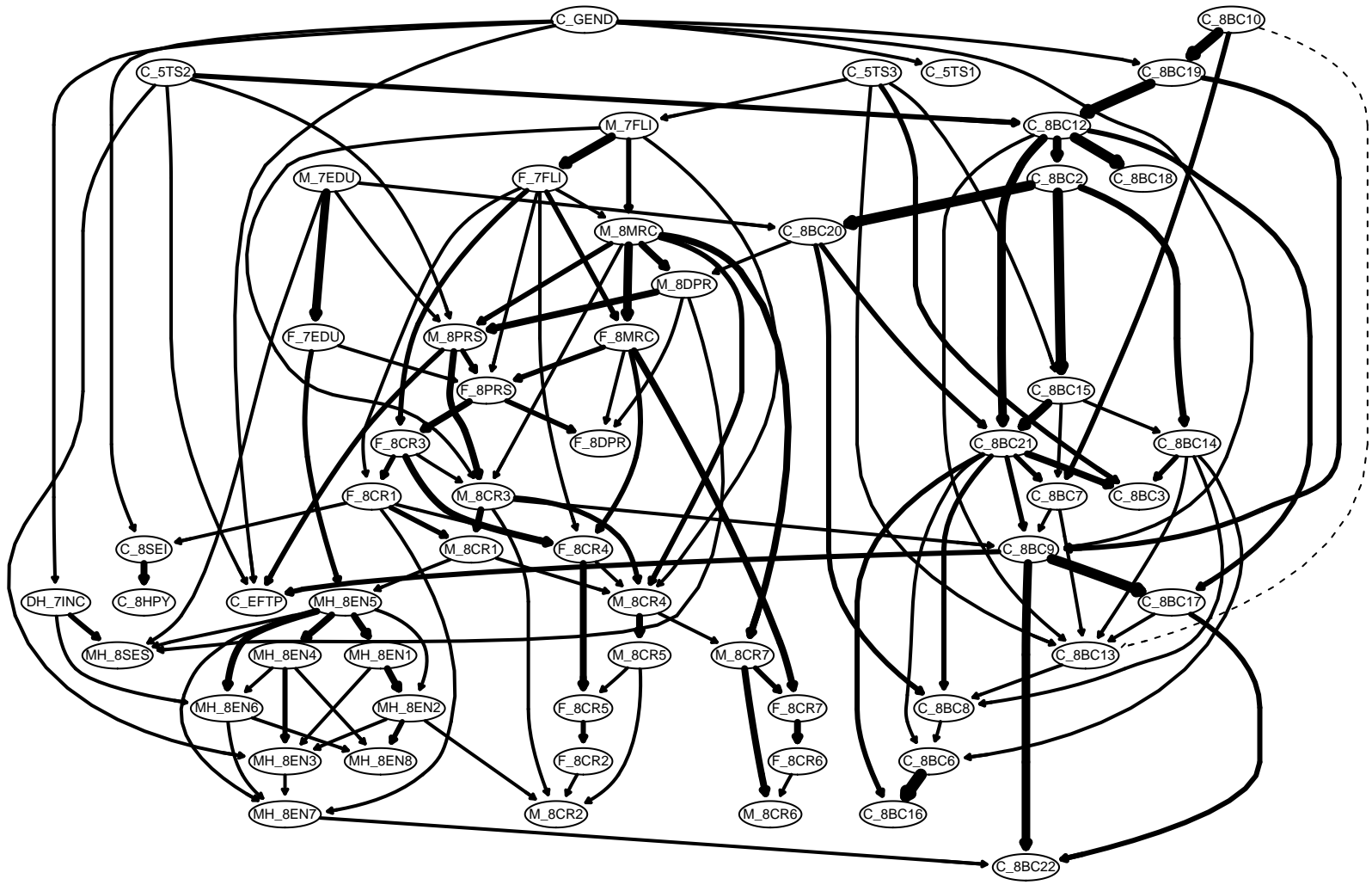

Supplement: Supplementary file 1 [file jintelligence-10-00074-s001.zip › Figure S17 Bayesian network structure in model 2 (threshold=.75).pdf]

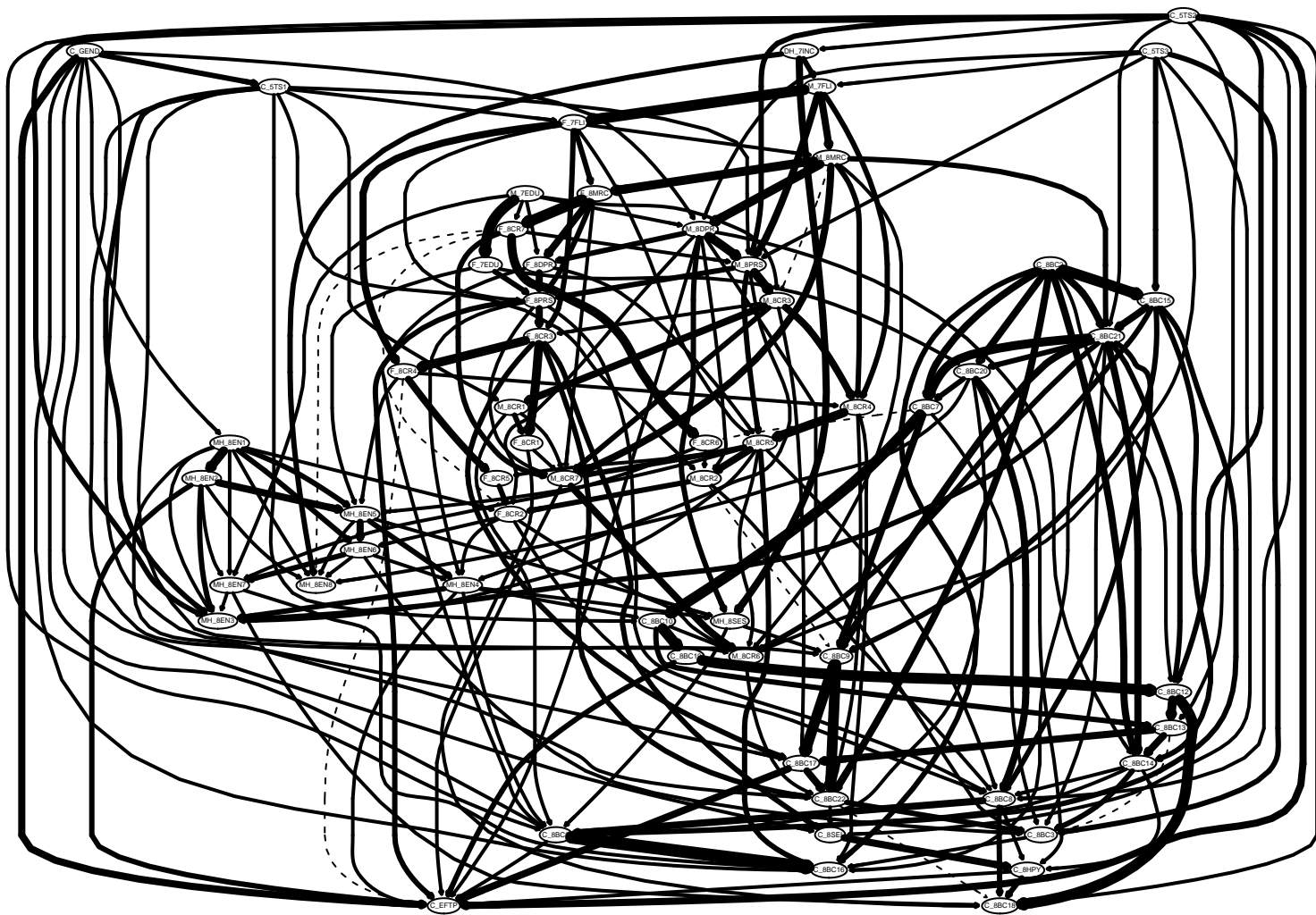

Supplement: Supplementary file 1 [file jintelligence-10-00074-s001.zip › Figure S18 Bayesian network structure in model 3 (threshold=.75).pdf]

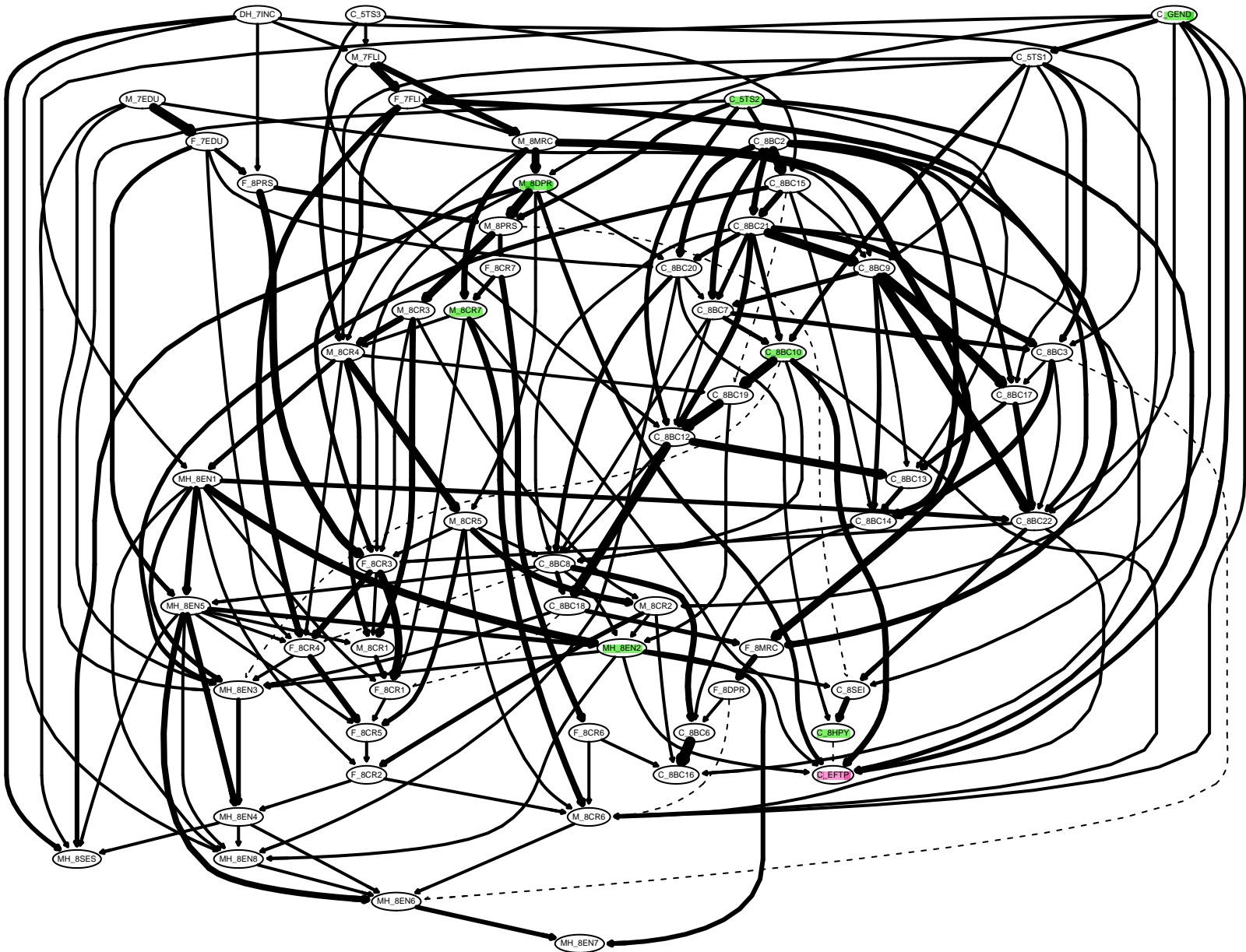

Supplement: Supplementary file 1 [file jintelligence-10-00074-s001.zip › Figure S19 Bayesian network structure in model 4 (threshold=.75).pdf]

averaged DAG

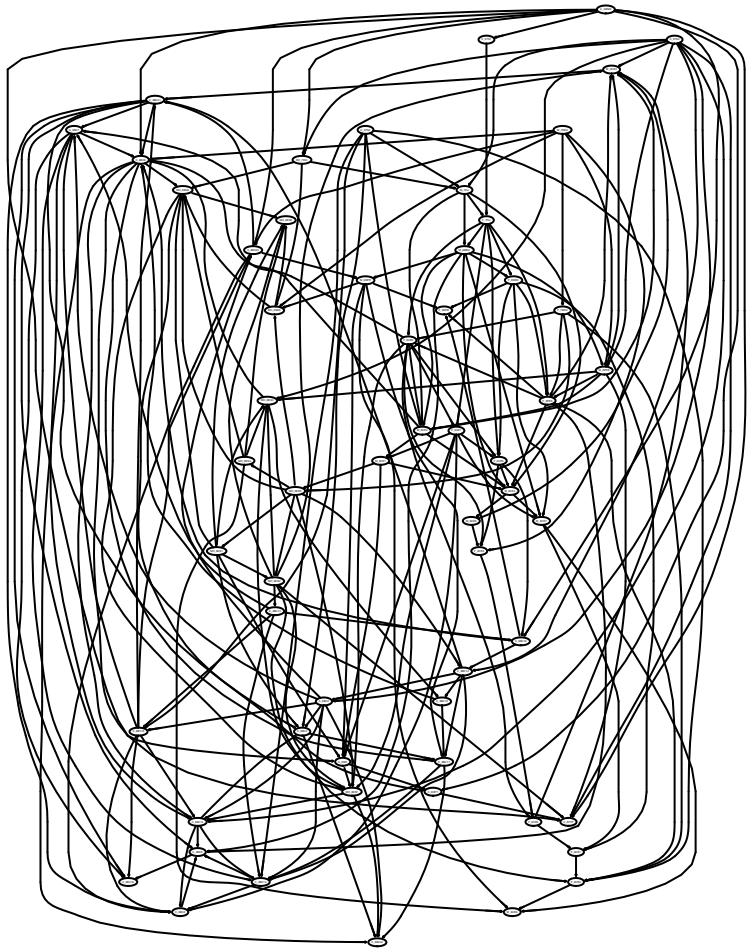

single DAG

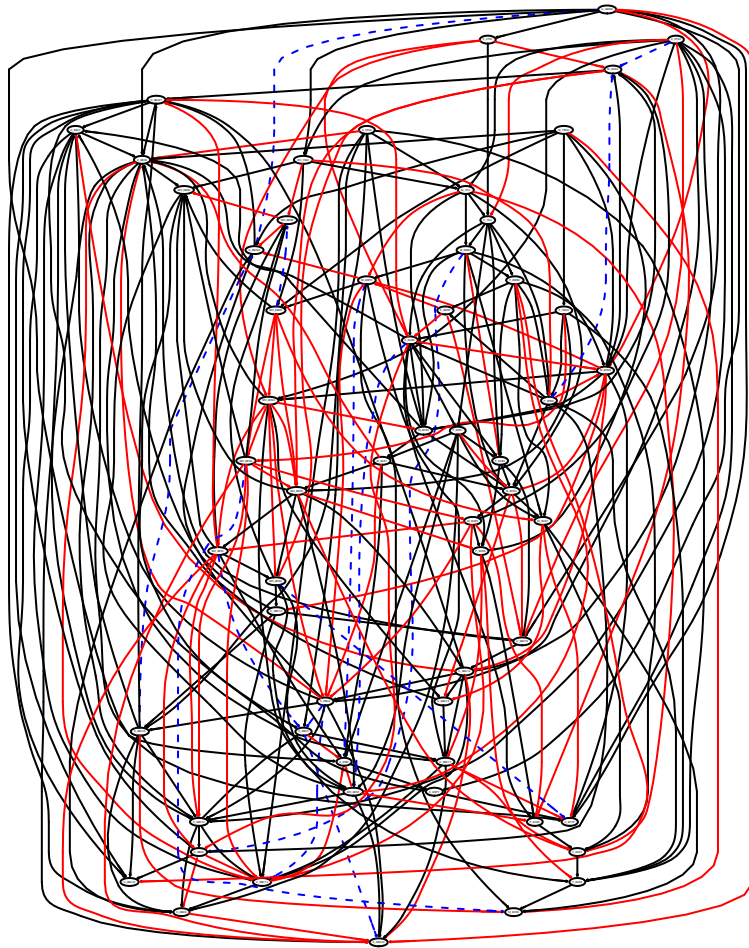

Supplement: Supplementary file 1 [file jintelligence-10-00074-s001.zip › Figure S2 DAG of bnlearning model 2.pdf]

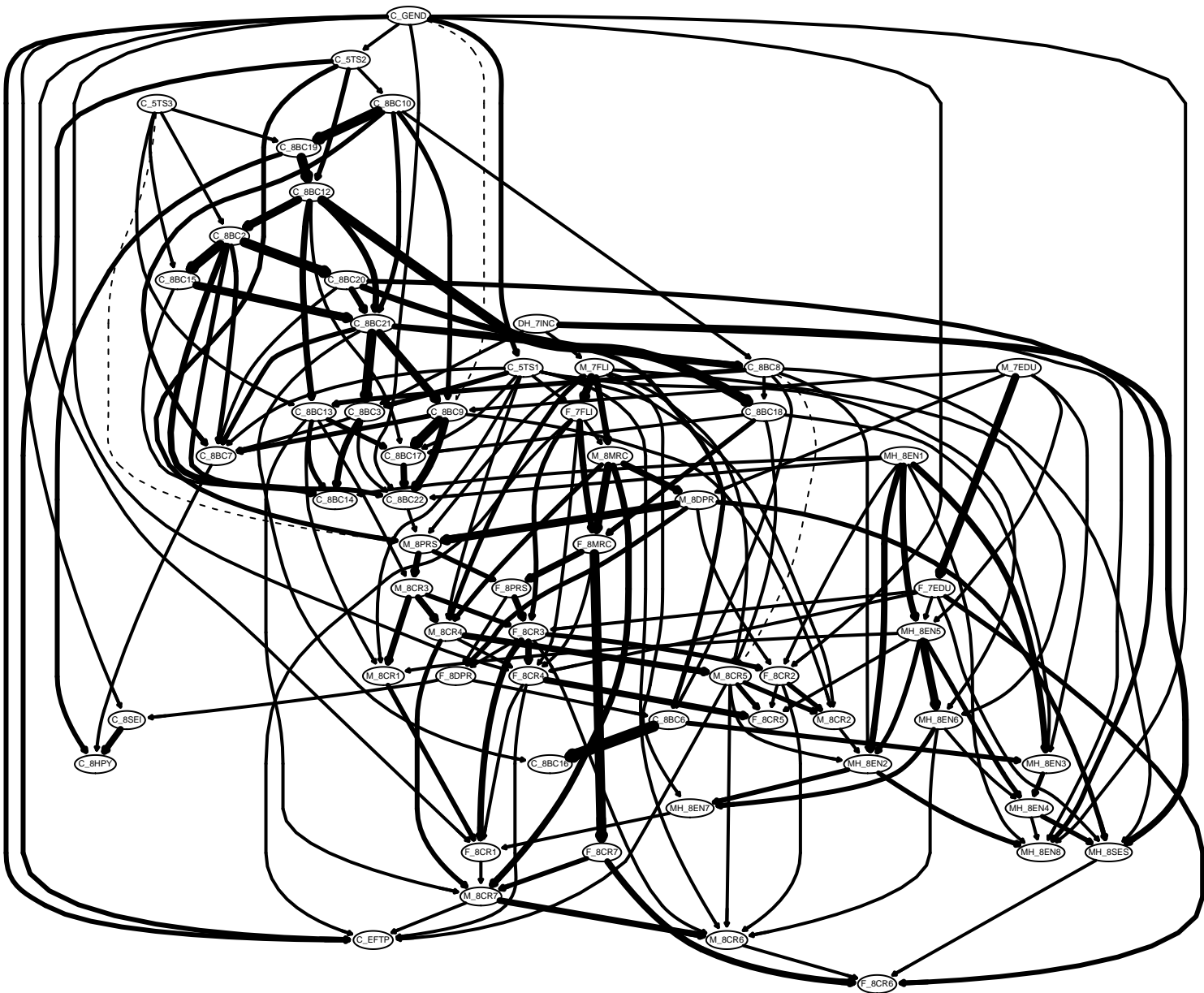

Supplement: Supplementary file 1 [file jintelligence-10-00074-s001.zip › Figure S20 Bayesian network structure in model 5 (threshold=.75).pdf]

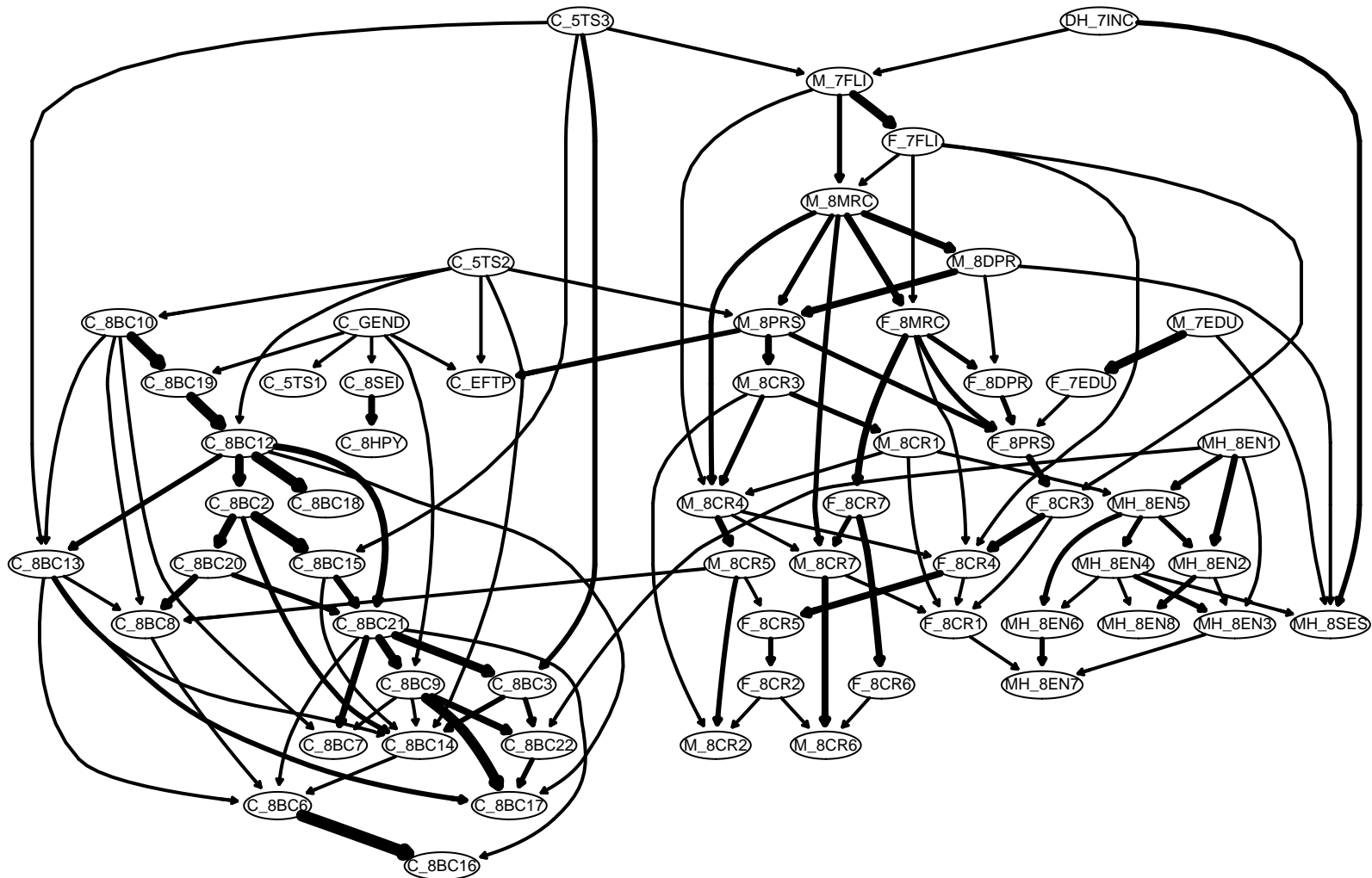

Supplement: Supplementary file 1 [file jintelligence-10-00074-s001.zip › Figure S21 Bayesian network structure in model 1 (threshold=.85).pdf]

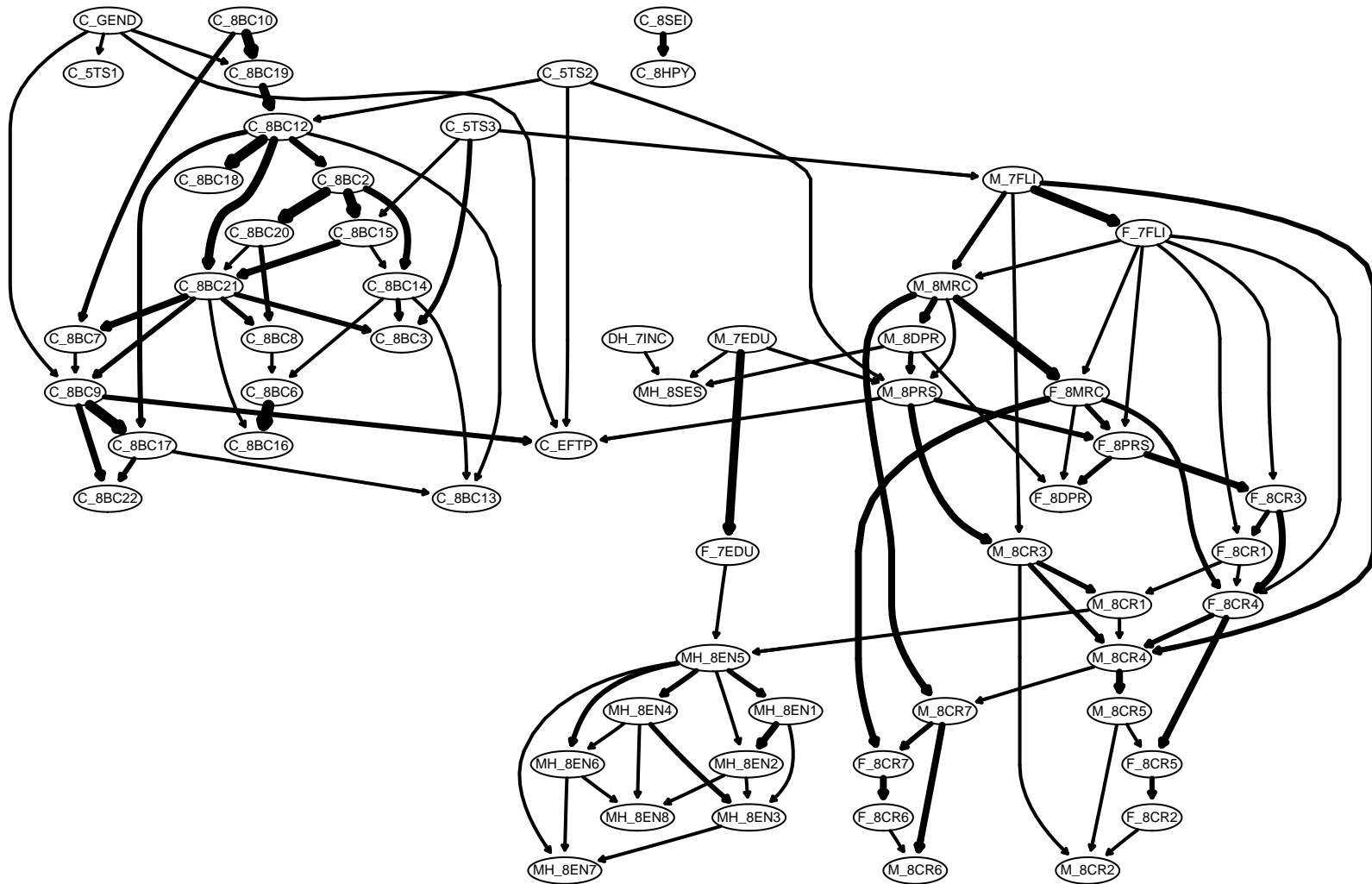

Supplement: Supplementary file 1 [file jintelligence-10-00074-s001.zip › Figure S22 Bayesian network structure in model 2 (threshold=.85).pdf]

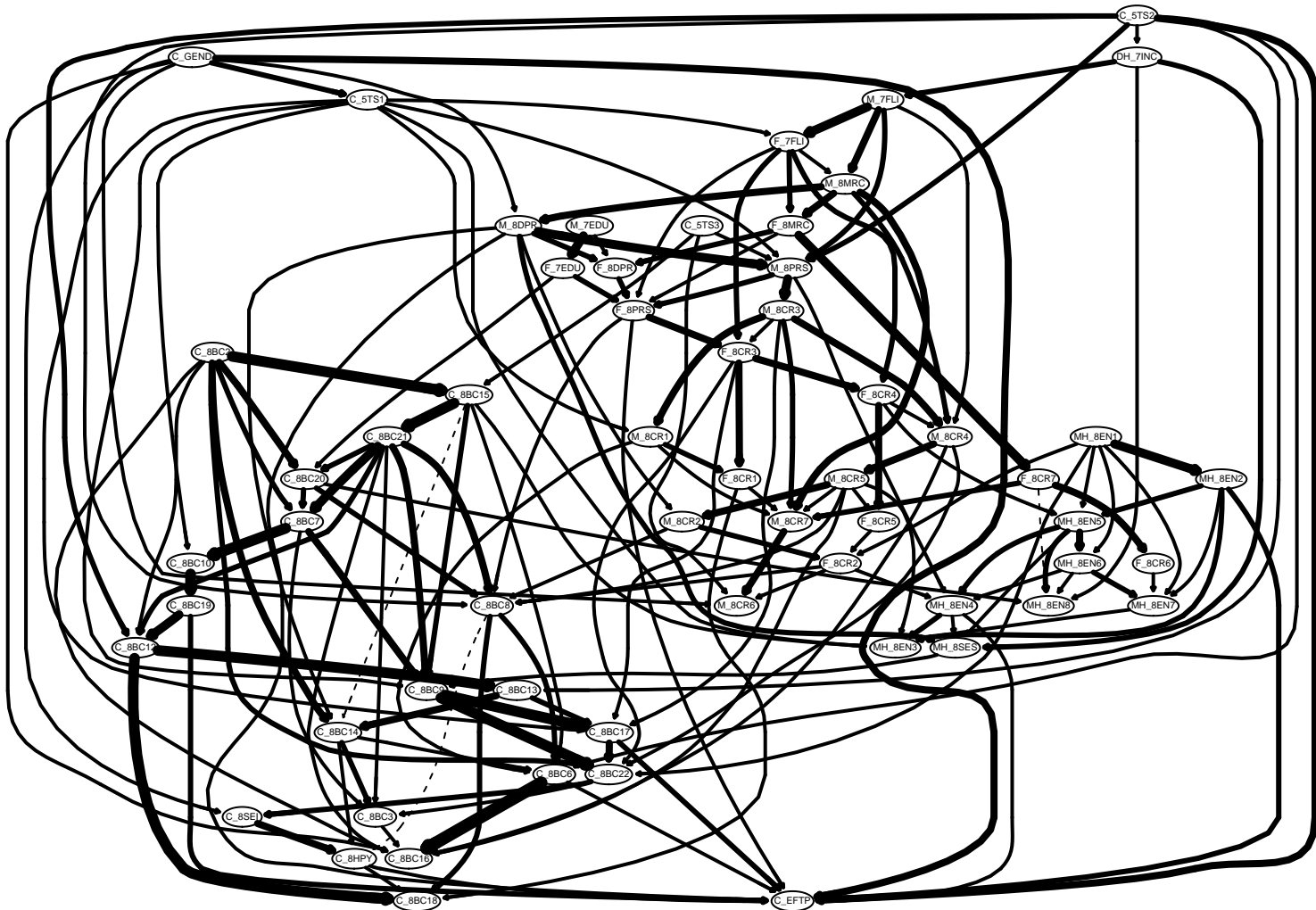

Supplement: Supplementary file 1 [file jintelligence-10-00074-s001.zip › Figure S23 Bayesian network structure in model 3 (threshold=.85).pdf]

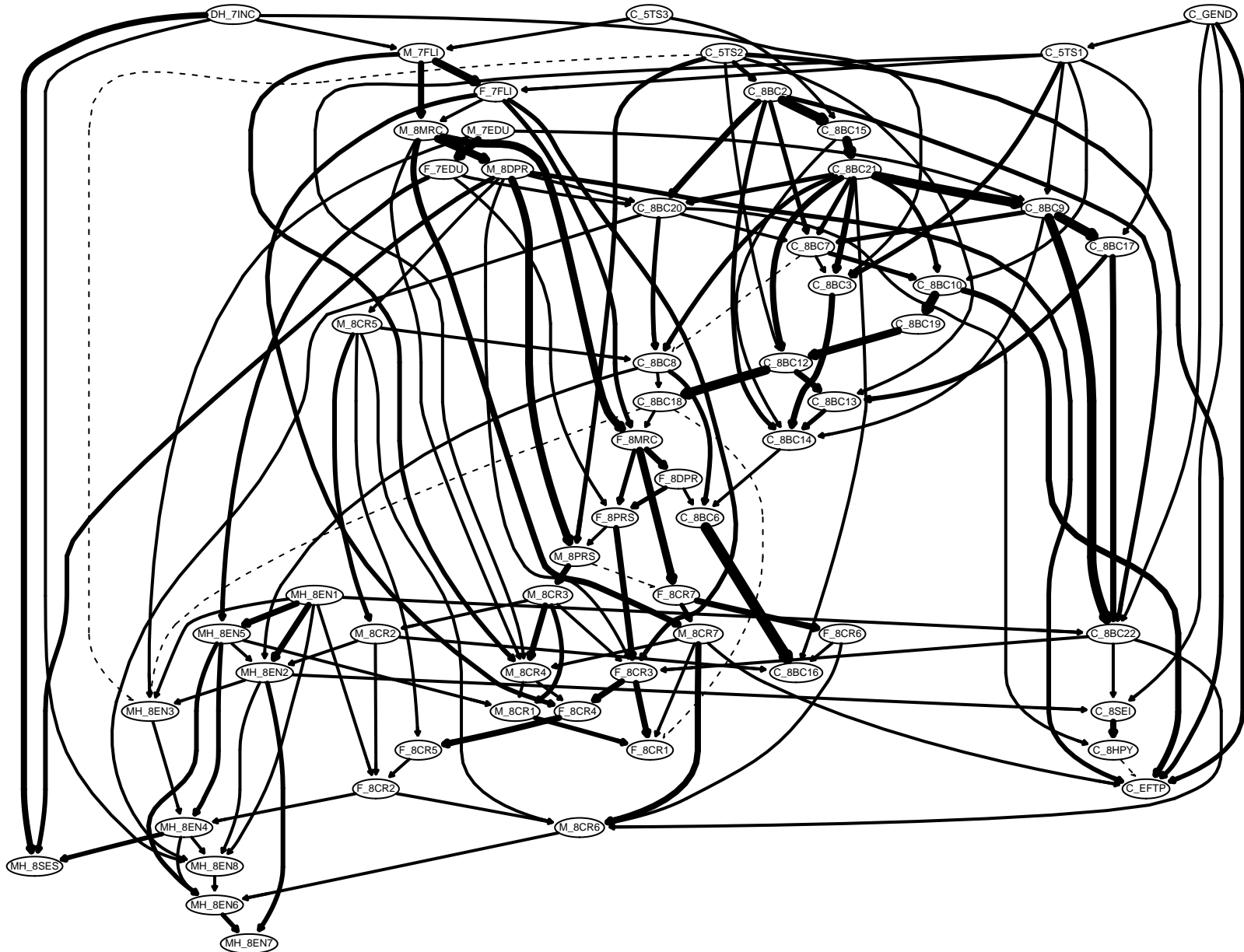

Supplement: Supplementary file 1 [file jintelligence-10-00074-s001.zip › Figure S24 Bayesian network structure in model 4 (threshold=.85).pdf]

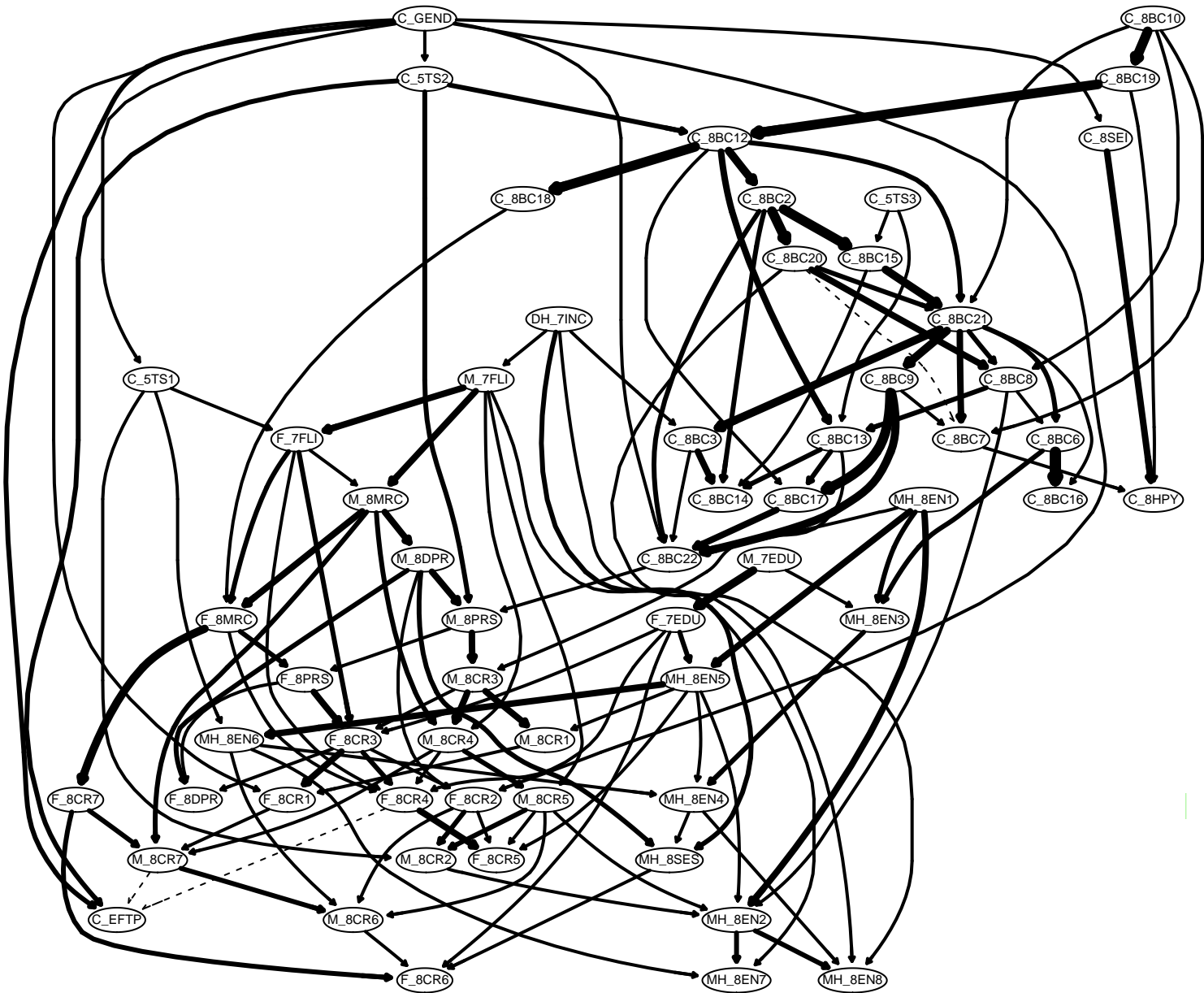

Supplement: Supplementary file 1 [file jintelligence-10-00074-s001.zip › Figure S25 Bayesian network structure in model 5 (threshold=.85).pdf]

Predictor

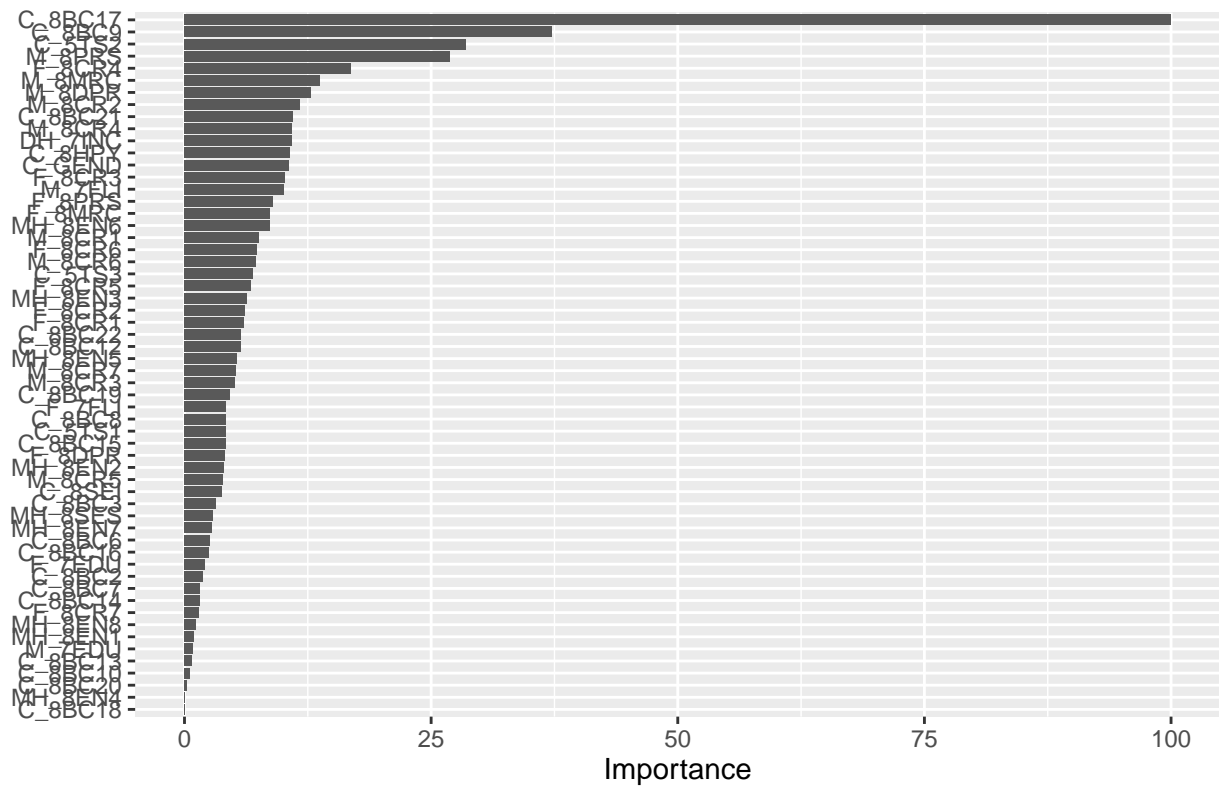

Supplement: Supplementary file 1 [file jintelligence-10-00074-s001.zip › Figure S26 Predictor in xgBoosting model 1.pdf]

Predictor

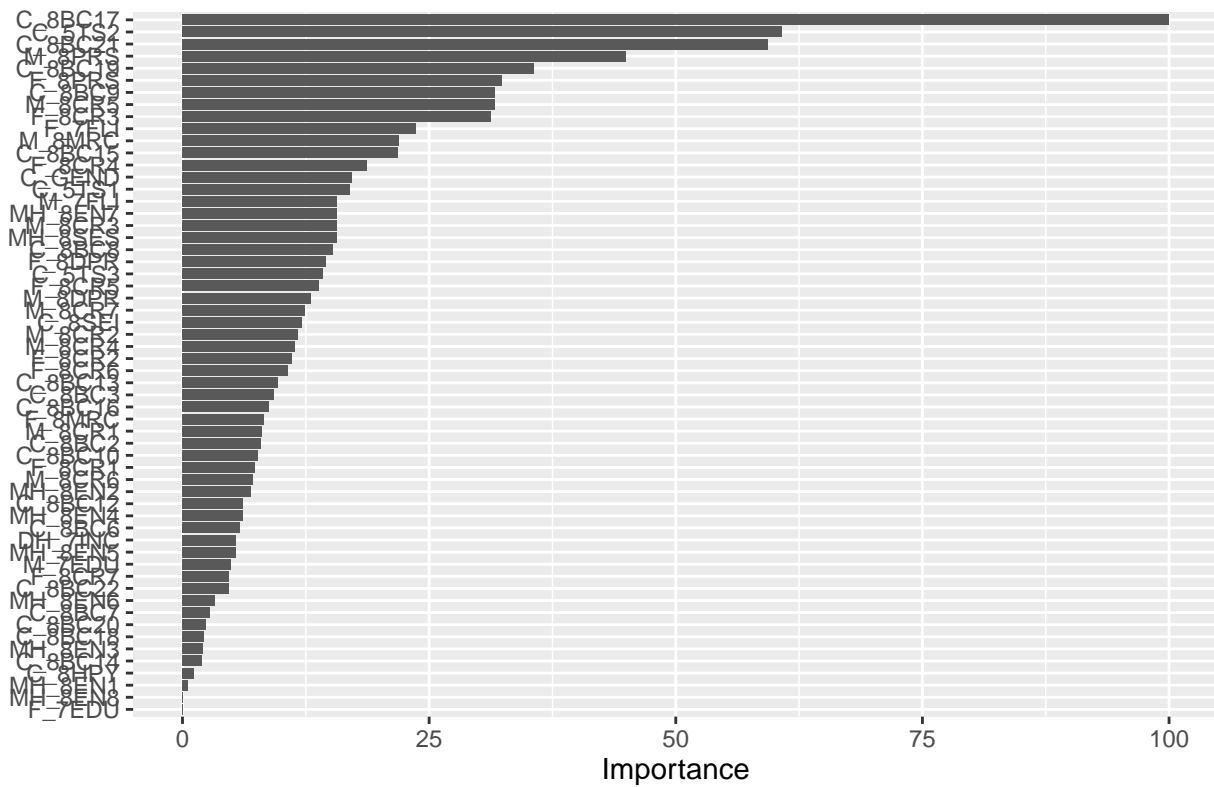

Supplement: Supplementary file 1 [file jintelligence-10-00074-s001.zip › Figure S27 Predictor in xgBoosting model 2.pdf]

Predictor

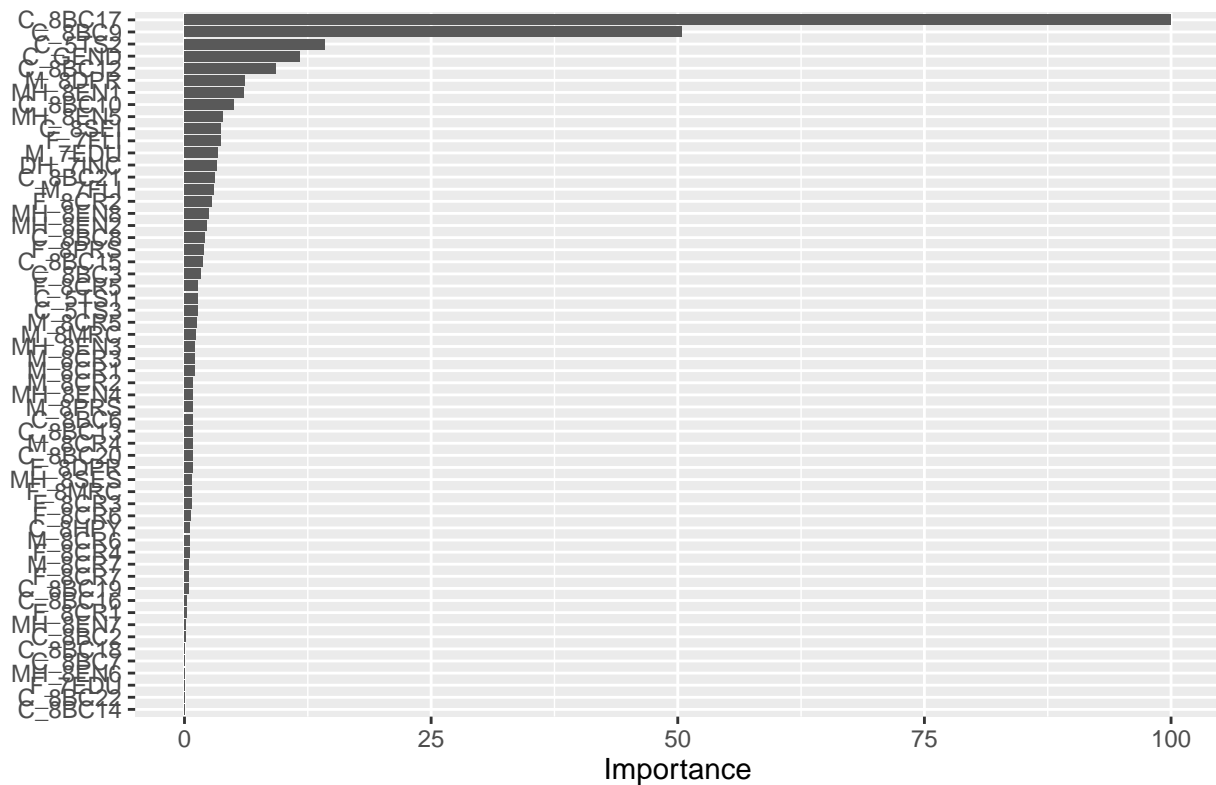

Supplement: Supplementary file 1 [file jintelligence-10-00074-s001.zip › Figure S28 Predictor in xgBoosting model 3.pdf]

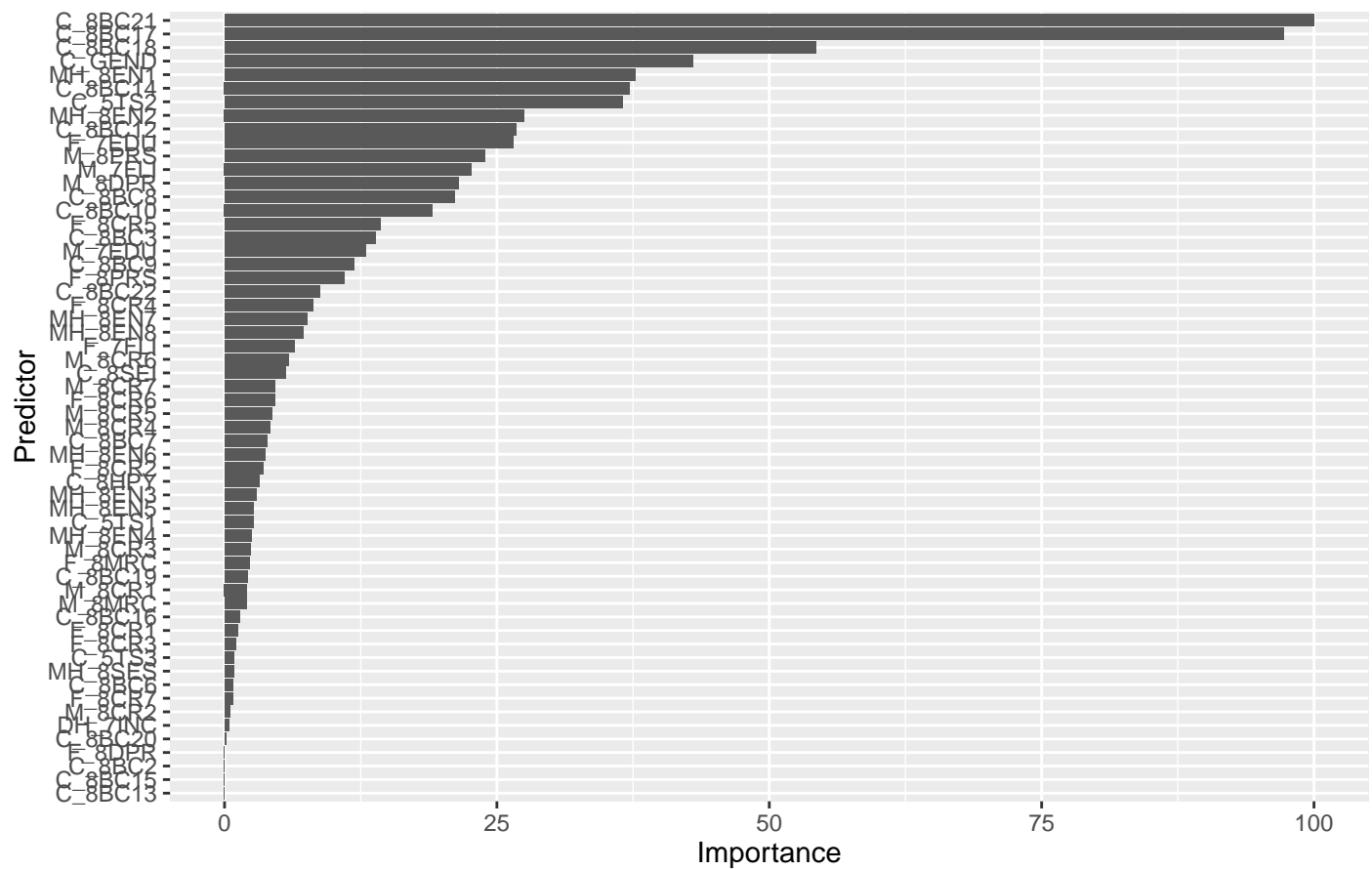

Supplement: Supplementary file 1 [file jintelligence-10-00074-s001.zip › Figure S29 Predictor in xgBoosting model 4.pdf]

averaged DAG

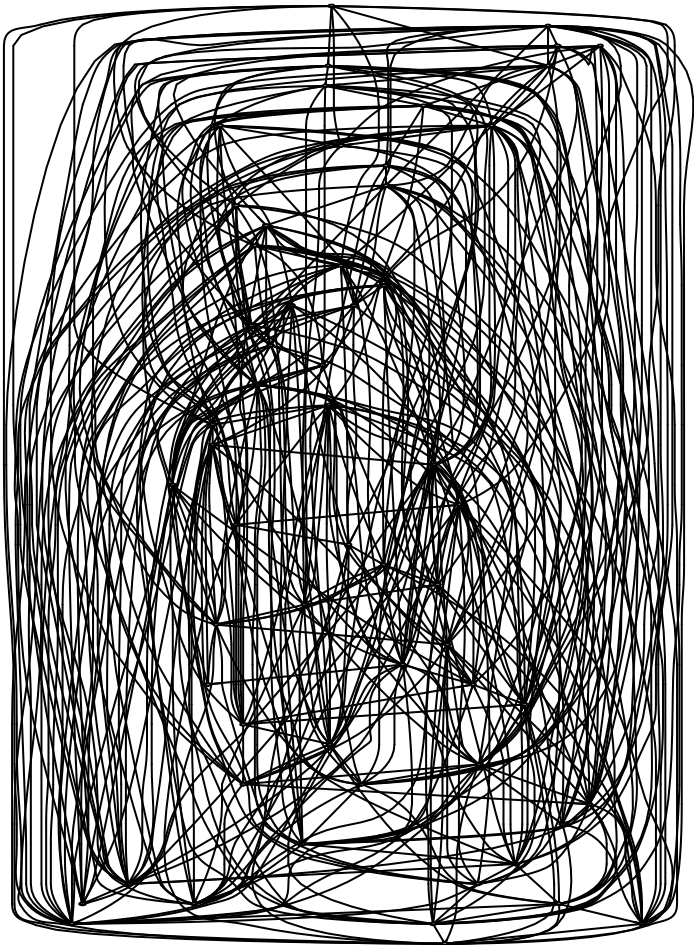

single DAG

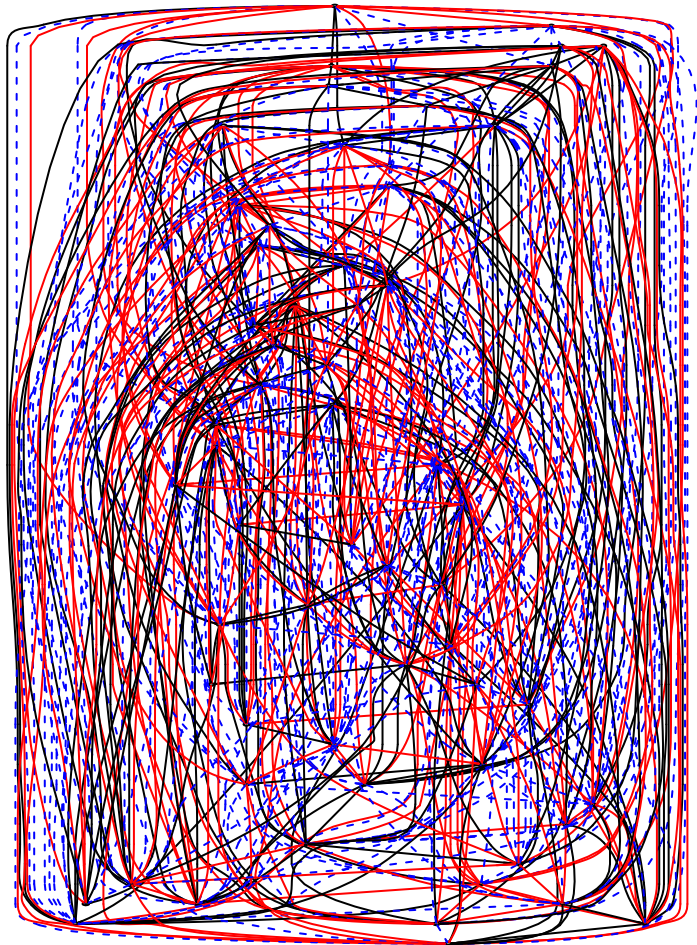

Supplement: Supplementary file 1 [file jintelligence-10-00074-s001.zip › Figure S3 DAG of bnlearning model 3.pdf]

Predictor

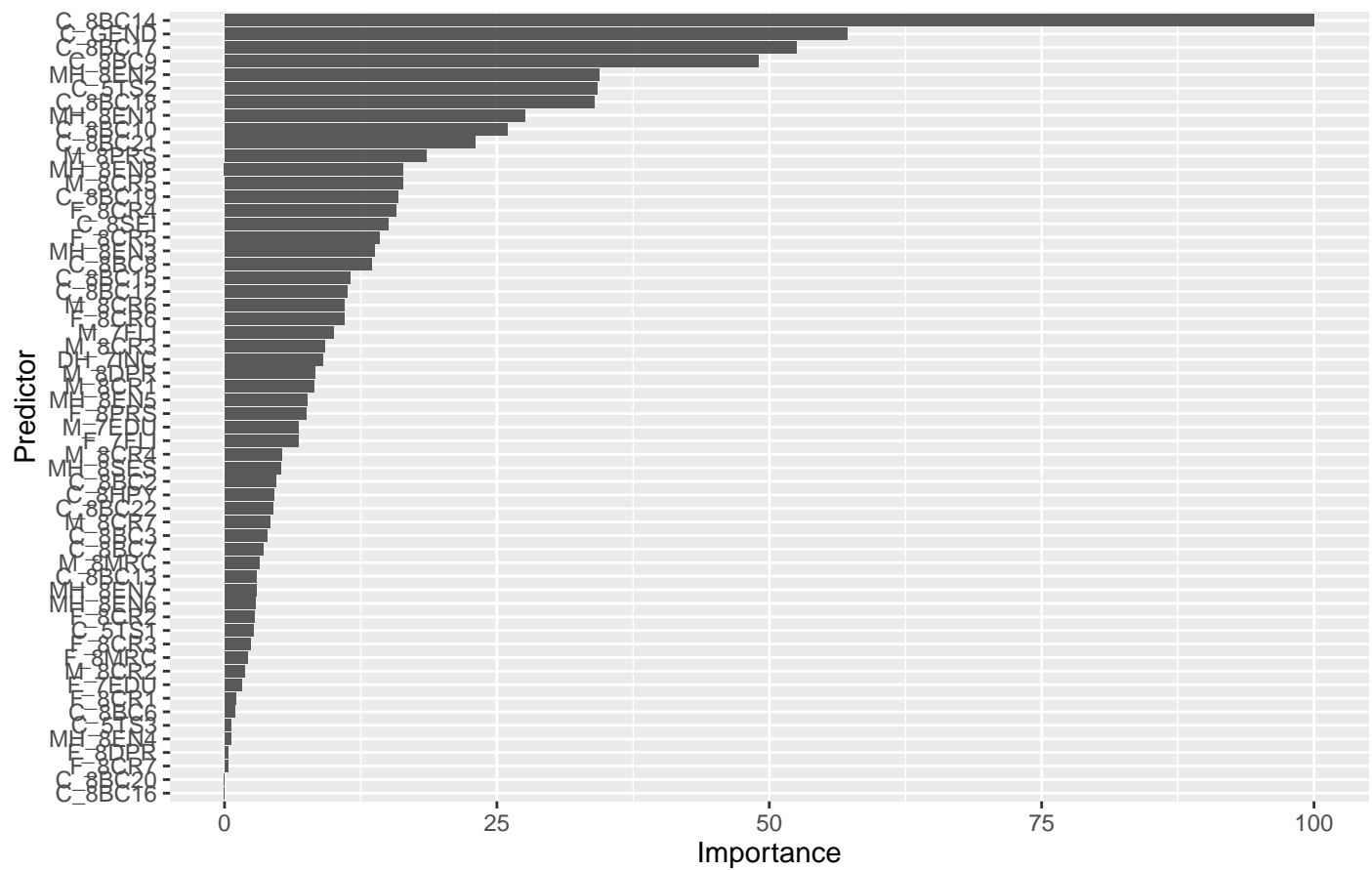

Supplement: Supplementary file 1 [file jintelligence-10-00074-s001.zip › Figure S30 Predictor in xgBoosting model 5.pdf]

averaged DAG

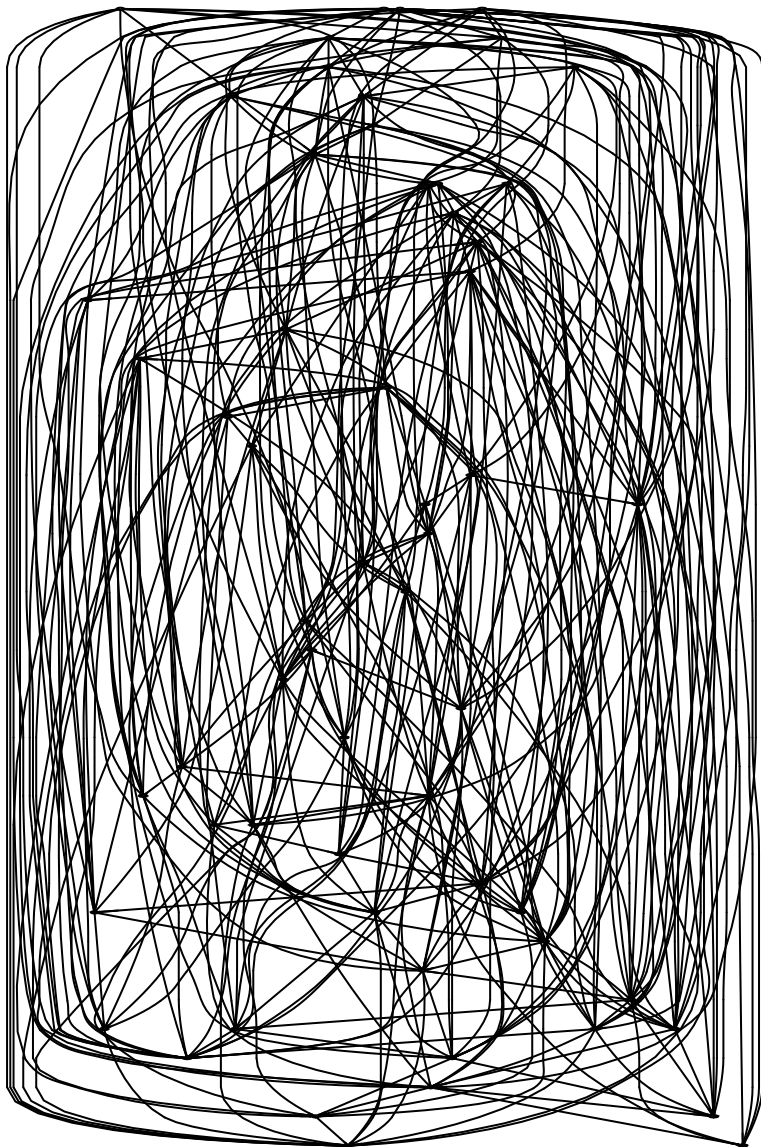

single DAG

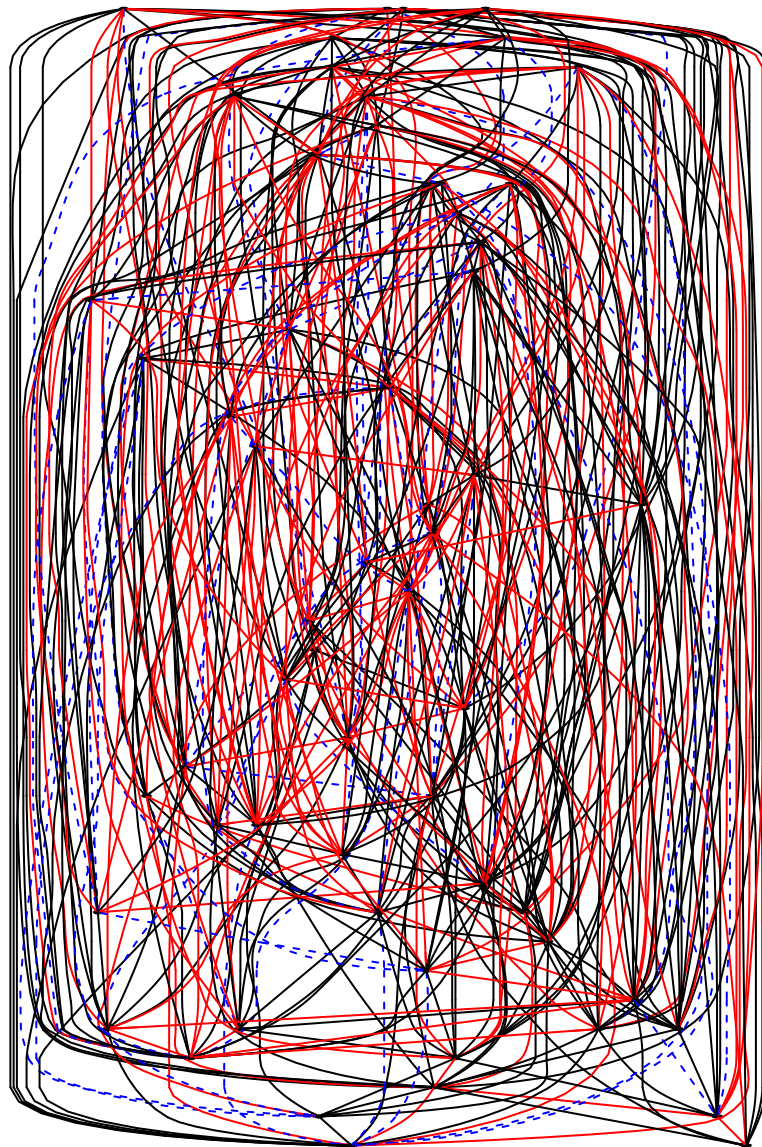

Supplement: Supplementary file 1 [file jintelligence-10-00074-s001.zip › Figure S4 DAG of bnlearning model 4.pdf]

averaged DAG

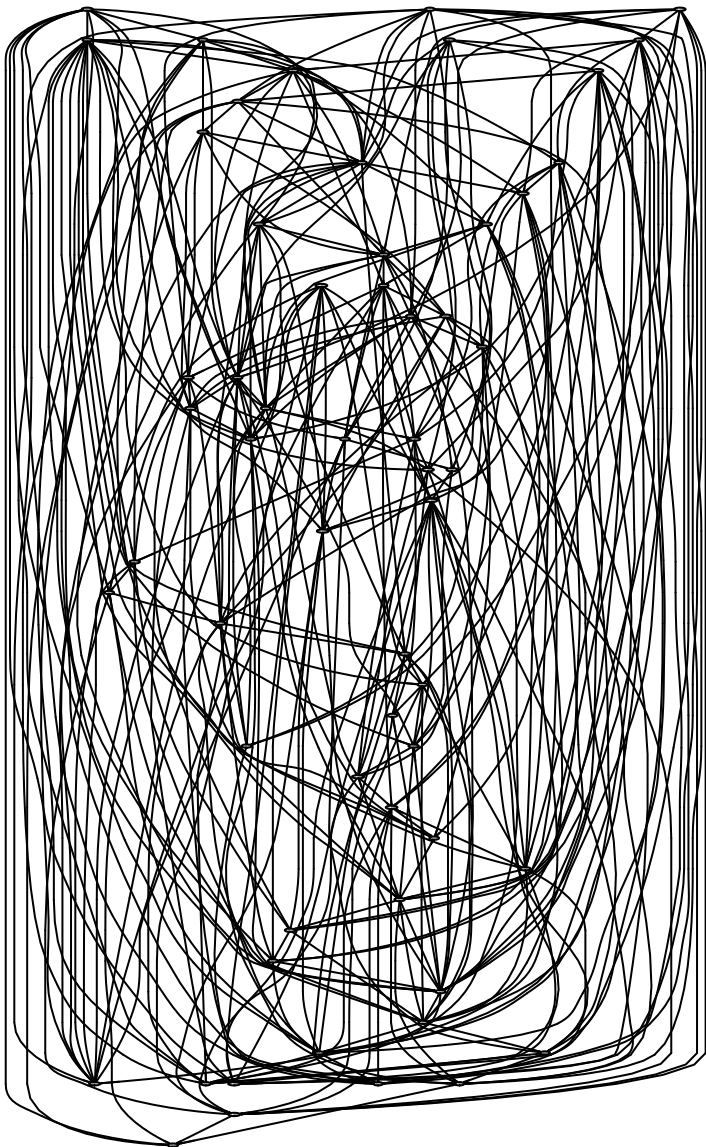

single DAG

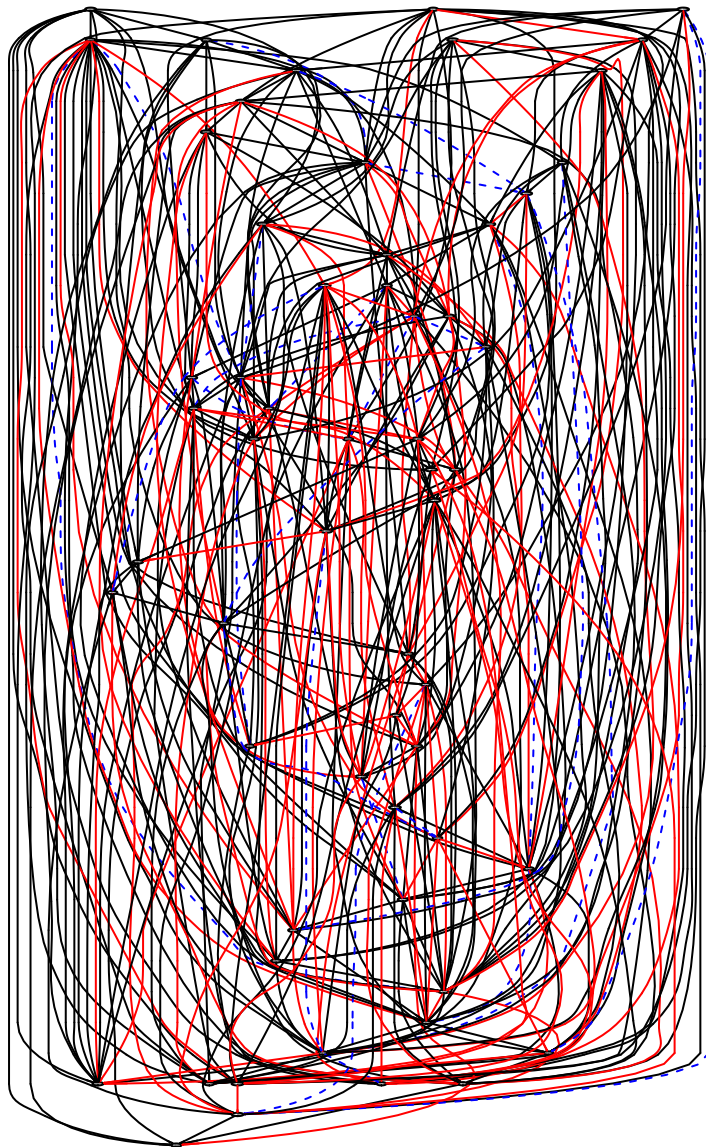

Supplement: Supplementary file 1 [file jintelligence-10-00074-s001.zip › Figure S5 DAG of bnlearning model 5.pdf]

**threshold = 0.501**

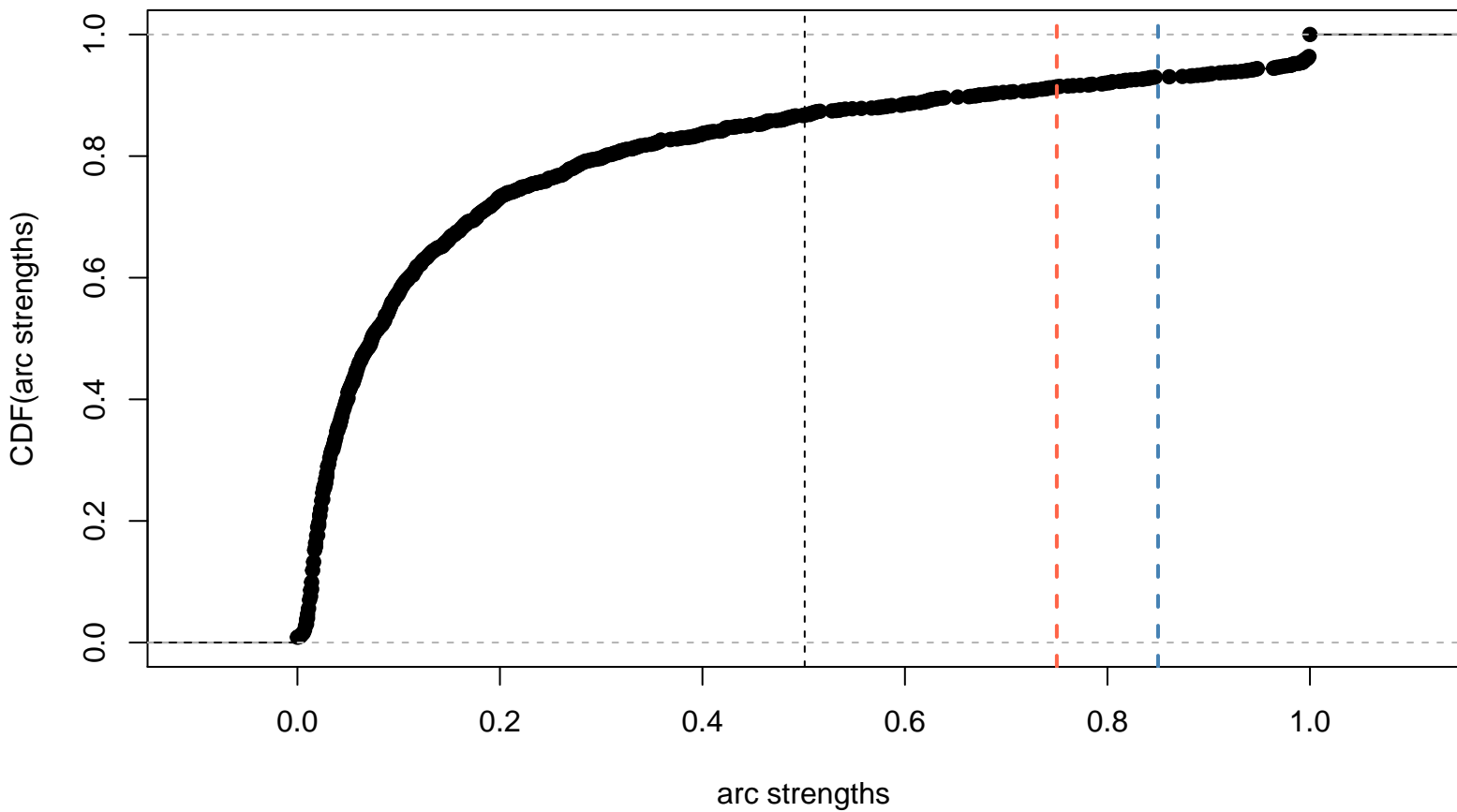

Supplement: Supplementary file 1 [file jintelligence-10-00074-s001.zip › Figure S6 Distribution of arc strengths in bnlearning model 1.pdf]

**threshold = 0.5**

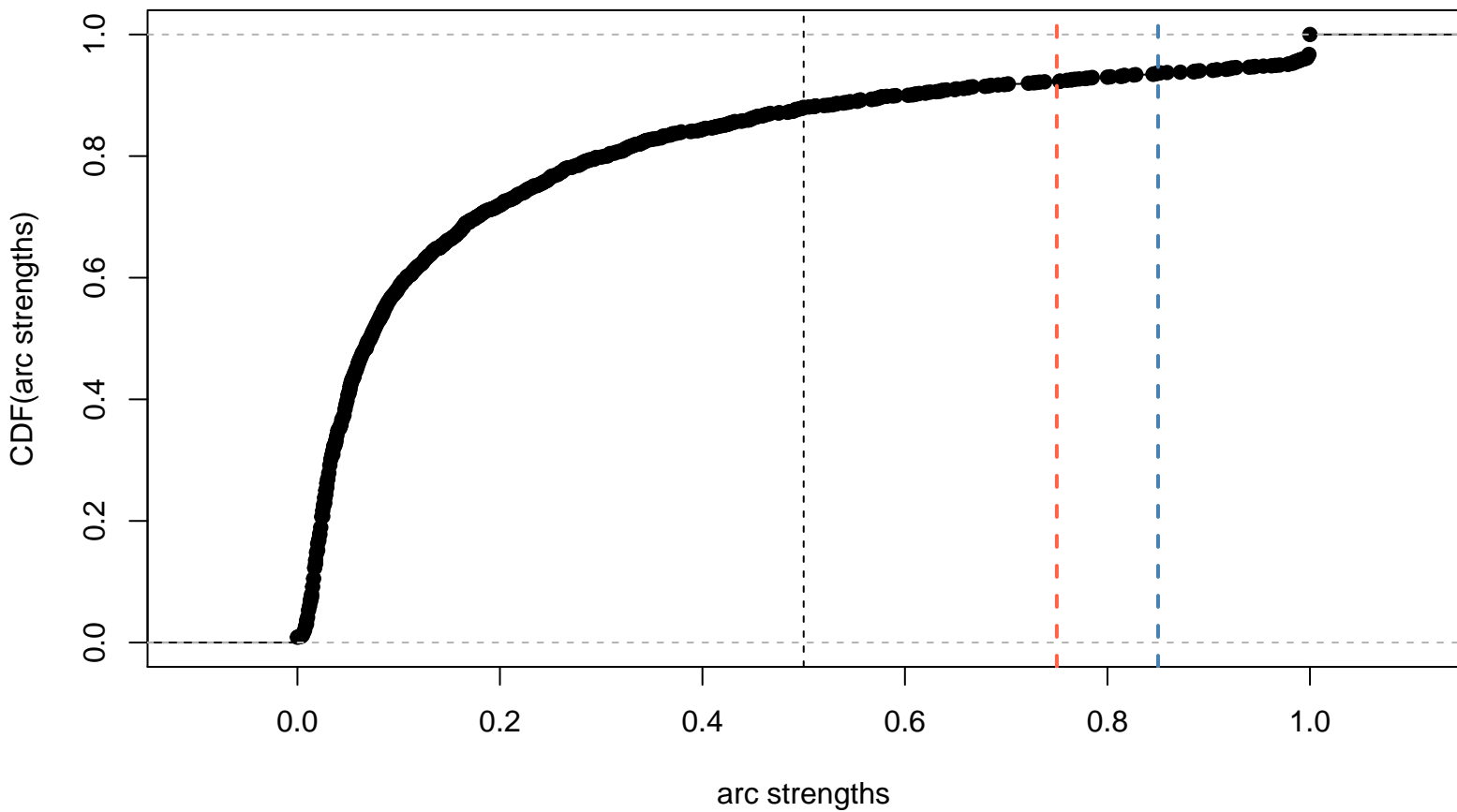

Supplement: Supplementary file 1 [file jintelligence-10-00074-s001.zip › Figure S7 Distribution of arc strengths in bnlearning model 2.pdf]

**threshold = 0.5**

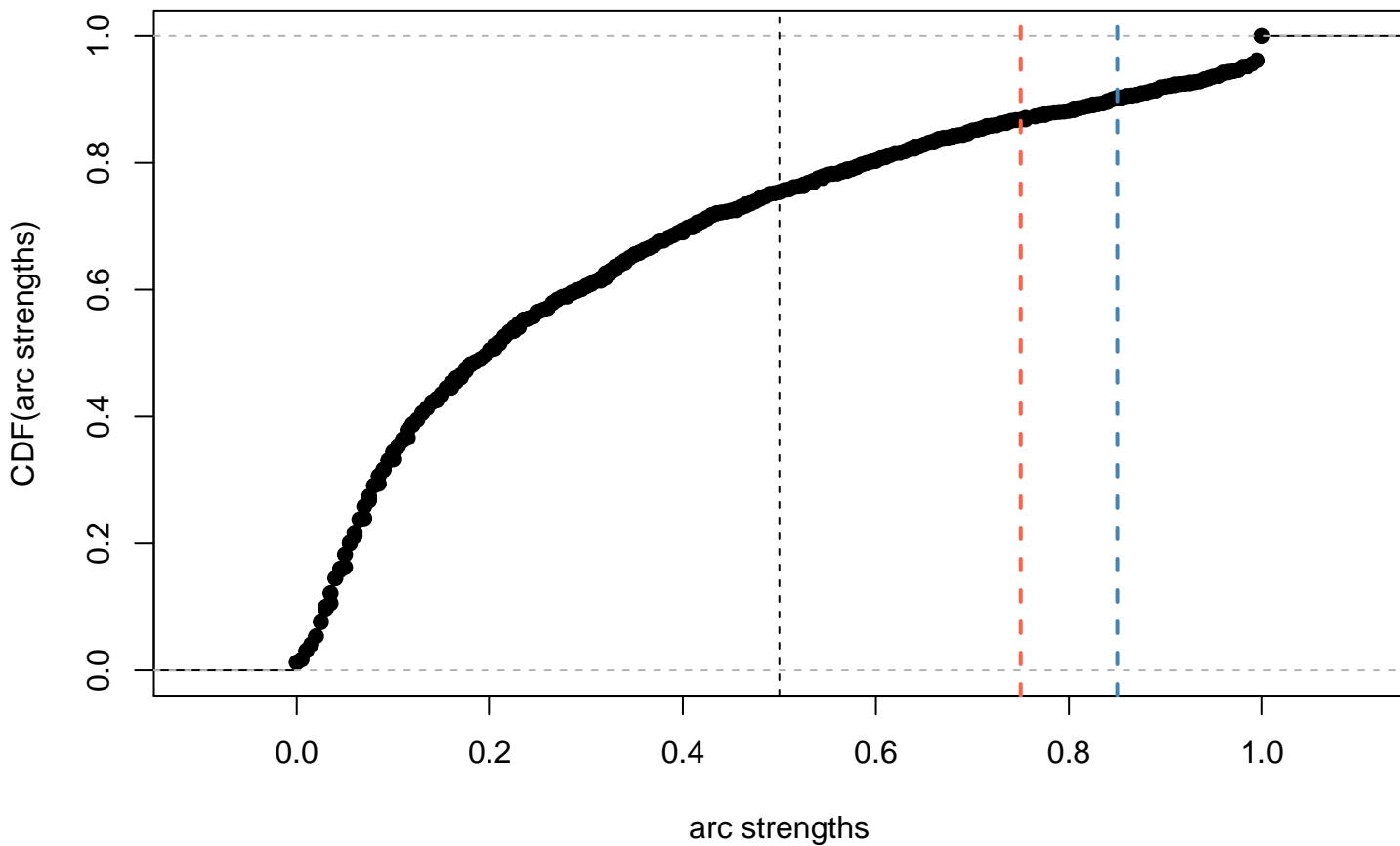

Supplement: Supplementary file 1 [file jintelligence-10-00074-s001.zip › Figure S8 Distribution of arc strengths in bnlearning model 3.pdf]

threshold = 0.498

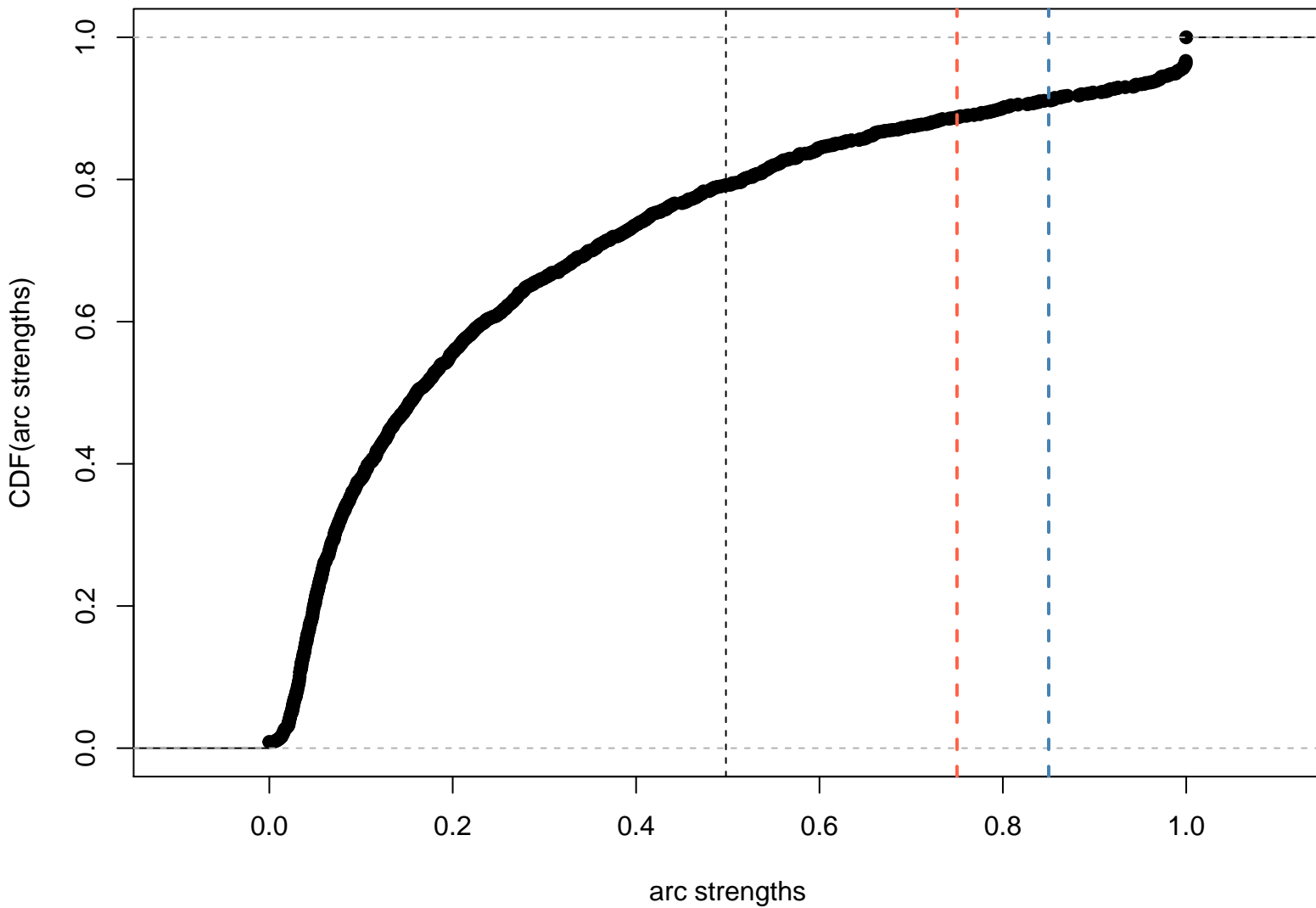

Supplement: Supplementary file 1 [file jintelligence-10-00074-s001.zip › Figure S9 Distribution of arc strengths in bnlearning model 4.pdf]
